# Accurate Delivery of Mesenchymal Stem Cell Spheroids With Platelet‐Rich Fibrin Shield: Enhancing Survival and Repair Functions of Sp‐MSCs in Diabetic Wound Healing

**DOI:** 10.1002/advs.202413430

**Published:** 2025-05-28

**Authors:** Jinglve Zhang, Wenqing Xu, Yutian Xiao, Dingheng Su, Yusheng He, Huohong Yang, Yixin Xie, Xiaofang Wang, Ren‐He Xu, Shaorong Lei, Dingyu Wu

**Affiliations:** ^1^ Department of Plastic Surgery Xiangya Hospital Central South University 87 Xiangya Road Changsha Hunan 410008 China; ^2^ National Clinical Research Center for Geriatric Disorders Xiangya Hospital Changsha Hunan 410008 China; ^3^ Xiangya School of Medicine Central South University Changsha 410083 China; ^4^ ImStem Biotechnology, Inc. 400 Farmington Avenue R1808 Farmington CT 06030 USA; ^5^ Zhuhai Hengqin ImStem Biotechnology Co., Ltd Hengqin New District Huandao Donglu 1889 Building 3 Zhuhai Guangdong 519000 China; ^6^ Ministry of Education Frontiers Science Center for Precision Oncology Center of Reproduction Development & Aging and Institute of Translational Medicine Faculty of Health Sciences University of Macau Taipa Macau China

**Keywords:** cell spheroid, diabetic wound, mesenchymal stem cell, platelet‐rich fiber, ROS protection

## Abstract

Diabetic wound is a significant clinical challenge, and stem cell therapy has shown great potential. This study explores the role of mesenchymal stem cell (MSC) spheroids (Sp‐MSCs) in healing diabetic wounds and the use of autologous plasma‐rich platelet fibrin (PRF) as a scaffold for Sp‐MSCs. Through activation of the coagulation system, PRF offers a protective fibrin shield for Sp‐MSCs to promote the rapid recovery migration and proliferation of MSCs while maintaining the activity of Sp‐MSCs in an inflammatory overload environment by activating the related genes of Integrin‐β1‐vascular endothelial growth factor (VEGF), and Wnt/β‐catenin pathways. The inclusion of Sp‐MSCs accelerates the gelation of PRF and results in improved mechanical strength. Additionally, PRF enhances the repair function of Sp‐MSCs, creating a favorable microenvironment for angiogenesis. In the wound model of diabetic mice, the combination of PRF with Sp‐MSCs accelerates wound healing. Results show that this combination significantly promotes wound repair and regulates the immune microenvironment. The study suggests that PRF is a promising bio‐derived scaffold for stem cell applications in diabetic wounds, offering new directions for stem cell therapy and biomimetic scaffold material development.

## Graphical abstract

Autologous plasma‐derived platelet‐rich fibrin (PRF) is prepared as a protective shield for mesenchymal stem cell spheroids (Sp‐MSCs). PRF forms a fibrin shield to protect Sp‐MSCs from the oxidative stress environment. The nutrients in PRF, particularly the α‐granules, can enhance the repair function of Sp‐MSCs. This opens up new possibilities for the clinical application of stem cells to treat diabetic wounds.

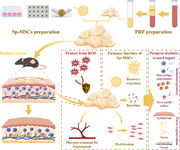

## 1 Introduction

Diabetes mellitus represents a burgeoning global health challenge, affecting an estimated 9.8% of the worldwide population.^[^ ^1^, ^2^ ^]^ A prevalent complication of this metabolic disorder is the development of diabetic wounds, which affect approximately 15% of individuals with diabetes and often lead to the formation of chronic, non‐healing ulcers.^[^ ^3^ ^]^ The prognosis for these ulcers is grim, with a long‐term mortality rate reaching 30%, and for those undergoing amputation, the rate soars to over 70%.^[^ ^4^ ^]^ Traditional therapeutic strategies, including self‐monitoring of blood glucose, wound debridement, flap transplantation, and revascularization, are often hindered by the complex microenvironment of diabetic wound beds, thereby limiting their efficacy in fostering wound healing.^[^ ^4^ ^]^ Evidence suggests that the protracted healing of diabetic ulcers is intricately linked to a paucity of mesenchymal stem cells (MSCs) at the wound site.^[^ ^5^ ^]^ This deficiency culminates in a dearth of essential growth factors and nutrients, which in turn amplifies the detrimental effects of inflammation and reactive oxygen species on the wound healing process.^[^ ^6^ ^]^

Consequently, mesenchymal stem cells (MSCs), endowed with the capacities for self‐renewal and multilineage differentiation, emerge as a potent therapeutic approach for the management of diabetic wounds.^[^ ^7^ ^]^ These cells foster tissue regeneration and cellular survival through their differentiation into dermal components, secretion of cytokines, and the release of extracellular vesicles, all of which contribute to the modulation of the inflammatory milieu.^[^ ^8^ ^]^ MSCs derived from human embryonic stem cells have illustrated their efficacy across a spectrum of disease models, attributable to their stable characteristics and amenability to large‐scale expansion.^[^ ^9^, ^10^, ^11^ ^]^ Moreover, MSC spheroids (Sp‐MSCs) demonstrate augmented capabilities in replicating in vivo conditions, tissue reparative processes, and immune modulation, owing to their three‐dimensional architecture and intensified intercellular interactions. This renders Sp‐MSCs a superior cell therapeutic strategy in the realm of regenerative medicine and tissue engineering.^[^ ^8^, ^12^ ^]^ Additionally, this approach is not only cost‐effective but also enhances the preservation, transportation, and the expedited restorative and healing attributes of the cells post‐transplantation.^[^ ^12^, ^13^ ^]^

In the context of diabetic wounds, the pathological microenvironment is characterized by elevated levels of inflammatory mediators and reactive oxygen species (ROS) that arise from hypoxia‐ischemia.^[^ ^14^ ^]^ Extensive research has demonstrated that the survival rate of engrafted stem cells is strikingly low, primarily due to the deleterious impact of inflammation and ROS.^[^ ^15^, ^16^, ^17^, ^18^ ^]^ These factors significantly compromise the functionality and viability of the transplanted mesenchymal stem cells (MSCs), thereby diminishing the therapeutic potency of stem cell transplantation.^[^ ^18^, ^19^, ^20^ ^]^ It is, therefore, imperative to establish a protective shield and nutrient reservoir for MSCs during the repair of diabetic wounds. This sanctuary is essential for shielding the cells from inflammation and ROS‐induced injury and for ensuring a continuous supply of nutrients and growth factors necessary for wound healing. Consequently, there is a pressing need for the development of a novel scaffold material capable of orchestrating and sustaining diverse bio‐microenvironments throughout the healing cascade, thereby augmenting the efficacy of stem cell therapies. Recently, a variety of bioactive carriers have been engineered and deployed in stem cell therapy. These carriers protect MSCs from the intricate and often hostile microenvironment present at disease sites, thereby empowering the cells to effectively execute their reparative functions in tissue regeneration.^[^ ^8^, ^18^ ^]^ Hydrogel materials have emerged as efficient vehicles for the delivery of MSCs; moreover, they have been shown to modulate the proliferative activity and stemness of these cells.^[^ ^21^ ^]^ Hence, there is a critical need to develop a stem cell carrier that could emulate the in vivo environment and closely follow the biological properties of the native tissue. Such a carrier would play a pivotal role in safeguarding and enhancing the recovery migration and in preserving the active functionality of Sp‐MSCs, which are crucial for effective regenerative medicine strategies.^[^ ^22^ ^]^ Platelet‐Rich Fibrin (PRF), a bioactive matrix derived from the lower layer of plasma following differential centrifugation of whole blood and subsequent thrombin activation.^[^ ^23^ ^]^ As a source of autologous plasma gel, PRF is widely used in the repair of various tissue injuries.^[^ ^24^ ^]^ Compared to synthetic materials, PRF is prepared on autologous tissue, and has no immune rejection reaction. Meanwhile, the fibrin network in PRF is closer to the tissue, and provides a good scaffold structure for tissue regeneration and cell adhesion at the site of wounds.^[^ ^25^ ^]^ The abundant growth factors contained in PRF play a positive role in inflammation regulation and promoting tissue regeneration which play important roles in accelerating and high‐quality tissue healing.^[^ ^26^ ^]^ Therefore, PRF has the potential to become a scaffold carrier for delivering stem cells for wound healing.

In the context of diabetic wound healing, the hostile microenvironment, replete with inflammatory cytokines and reactive oxygen species (ROS) stemming from hypoxia‐ischemia, poses a significant challenge for stem cell survival. To counteract this, the present study introduces We have developed a novel approach by coating Spheroid Mesenchymal Stem Cells (Sp‐MSCs) with PRF gel, thereby creating a protective shield around Sp‐MSCs. This PRF‐coated Sp‐MSC construct is anticipated to withstand the ROS‐mediated assault in the inflammatory milieu characteristic of diabetic wounds, while also providing a biomimetic scaffold that facilitates Sp‐MSCs colonization and adherence within the wound bed. This strategy is designed to preserve the viability and enhance the reparative functions of Sp‐MSCs.

## 2 Result

### 2.1 The Fabrication and Characterization of Sp‐MSCs

**Figure 1:**
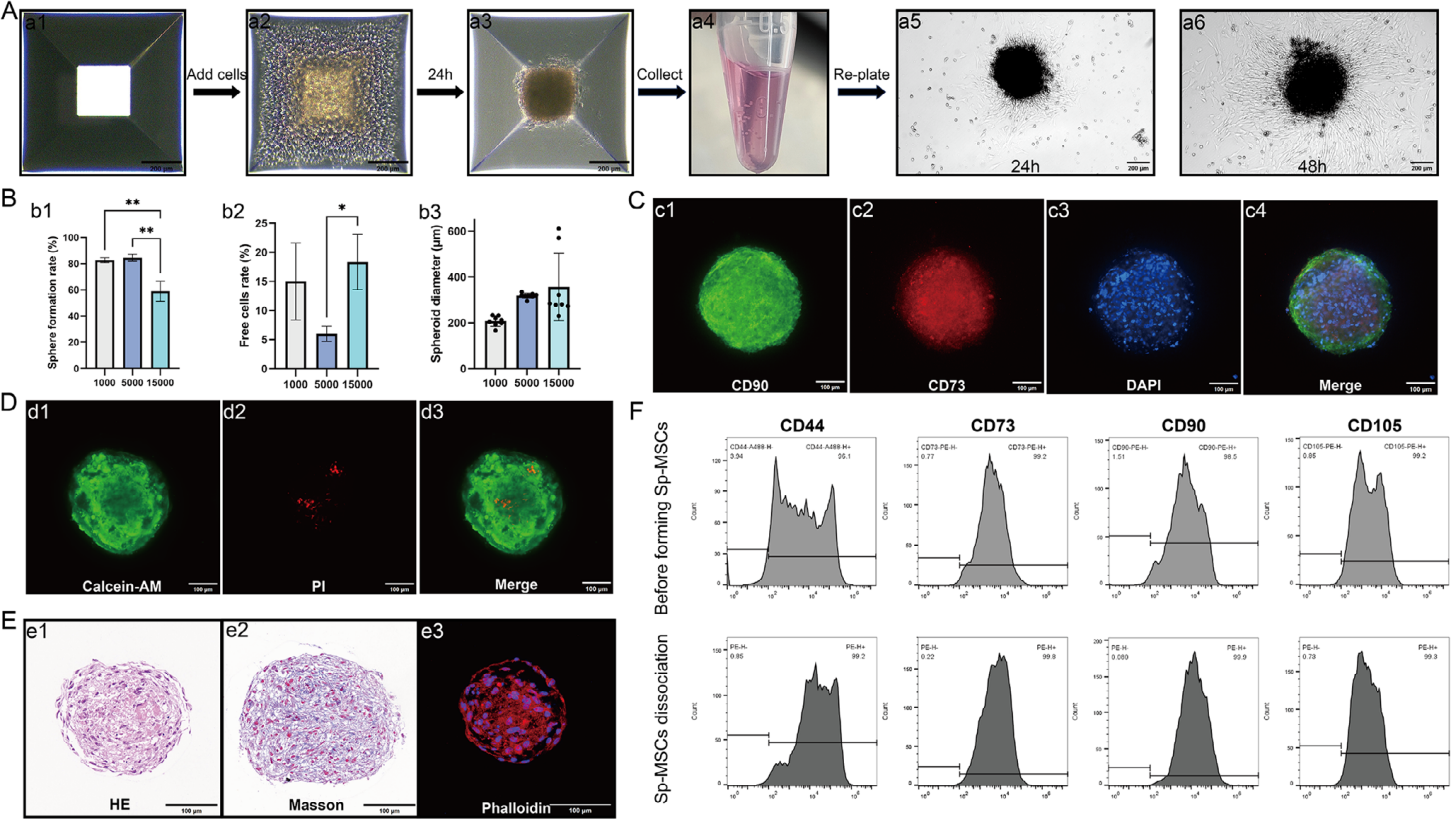
The fabrication and characterization of Sp‐MSCs. A) The process of cell spheroids forming plate method for Sp‐MSCs. a1–a4) Add MSCs into cell spheroids forming plate wells, spheroids forming, Sp‐MSCs collection. a5,a6) Image of Sp‐MSCs recovery and crawling out. (Scale bar = 200 µm). B) 5000 cells/spheroid had the most stable structure and the highest sphere formation rate. b1) sphere formation rate of different sizes Sp‐MSCs (*n* = 3), b2) Free cells rate of different sizes Sp‐MSCs (*n* = 3), b3) The diameters of different sizes Sp‐MSCs (*n* = 8), (*p < 0.05, **p < 0.01). C) Stemness unchanged in Sp‐MSCs. Stem cell markers (CD90, green; CD73, red; DAPI, blue) of the cells in the Sp‐MSCs (Scale bar = 100 µm). D)The microscopy images of live/dead stained Sp‐MSCs after 3 days in vitro culture showed no significant cell death in Sp‐MSCs (Scale bar = 100 µm). E) H&E staining, Masson staining, Phalloidin staining of Sp‐MSCs (Scale bar = 100 µm). F) Flow cytometry of CD44, CD73, CD90, and CD105 of MSCs before forming Sp‐MSCs, and Sp‐MSCs dissociate into individual cells.

Here, we use the cell spheres forming plate to prepare mesenchymal stem cell spheroids (Sp‐MSCs) (**Figure** **1**A,a1–a4). To detect Sp‐MSCs activity, we re‐plate Sp‐MSCs onto the culture plate. MSCs in Sp‐MSCs recover and crawl out of the spheroids (Figure 1A,a5,a6). By controlling the cell seeding density, Sp‐MSCs expected to contain different cells (1000/5000/15 000) could be prepared. The results showed that 1000/5000 cells/spheroid had a higher spheroid formation rate than 15 000 cells/spheroid (Figure 1B,b1). And 5000 cells/spheroid had a lower free cell rate than the other groups (Figure 1B,b2). By measuring the diameter of Sp‐MSCs, we found that 5000 cells/spheroid had the most stable spherical diameter (318.57 ± 10.99 µm). The diameter of 1000 cells/spheroid was smaller (207.08 ± 20.82 µm), and more free cells can be seen in wells. The 15 000 cells/spheroid had a large number of free cells and the Sp‐MSCs had an uneven diameter (356.07 ± 137.85 µm) (Figure 1B,b3; Figure S1A, Supporting Information). Overall, 5000 cells/spheroid exhibited the most stable spherical form and the highest cell spheroidization rate. Therefore, we chose 5000 cells/spheroid for our subsequent experiments. The live/dead staining revealed that the Sp‐MSCs were stored in MSC culture medium for 3 days at 25 °C; the vast majority of cells survived, with only a tiny percentage of cells dying in the middle (Figure 1D; Figure S1B, Supporting Information). MSC surface antibodies characterized were evaluated using immunofluorescence and flow cytometry to verify the effect of Sp‐MSCs formation on stem cell stemness. MSCs markers CD73/CD90 remain highly positive in the sphere state of cells (Figure 1C; Figure S1C, Supporting Information). Flow cytometry results revealed that compared to MSC, the expression levels of stem cell surface antibodies CD44, CD73, CD90, and CD105, and did not show significant changes after Sp‐MSCs dissociated into individual MSC (Figure 1F). After the formation of Sp‐MSCs, stem cells aggregated into clusters with stable structures and are not easily loose. Sp‐MSCs slice staining and cytoskeleton staining showed that the stem cells in Sp‐MSCs were tightly bound (Figure 1E). Sp‐MSCs were stored in an MSC culture medium at 25 °C, and the diameter of Sp‐MSCs gradually decreased with the extension of storage days (Figure S1D, Supporting Information). After 7 days of storage, the diameter of Sp‐MSCs decreased to around 200 µm, and the difference of Sp‐MSCs’ diameter increased (Figure S1E, Supporting Information). Sp‐MSCs can still recover and crawl out after 7 days of storage (Figure S1F, Supporting Information).

### 2.2 PRF Combined with Sp‐MSCs, Accelerated Coagulation, and Enhanced Strength

**Figure 2:**
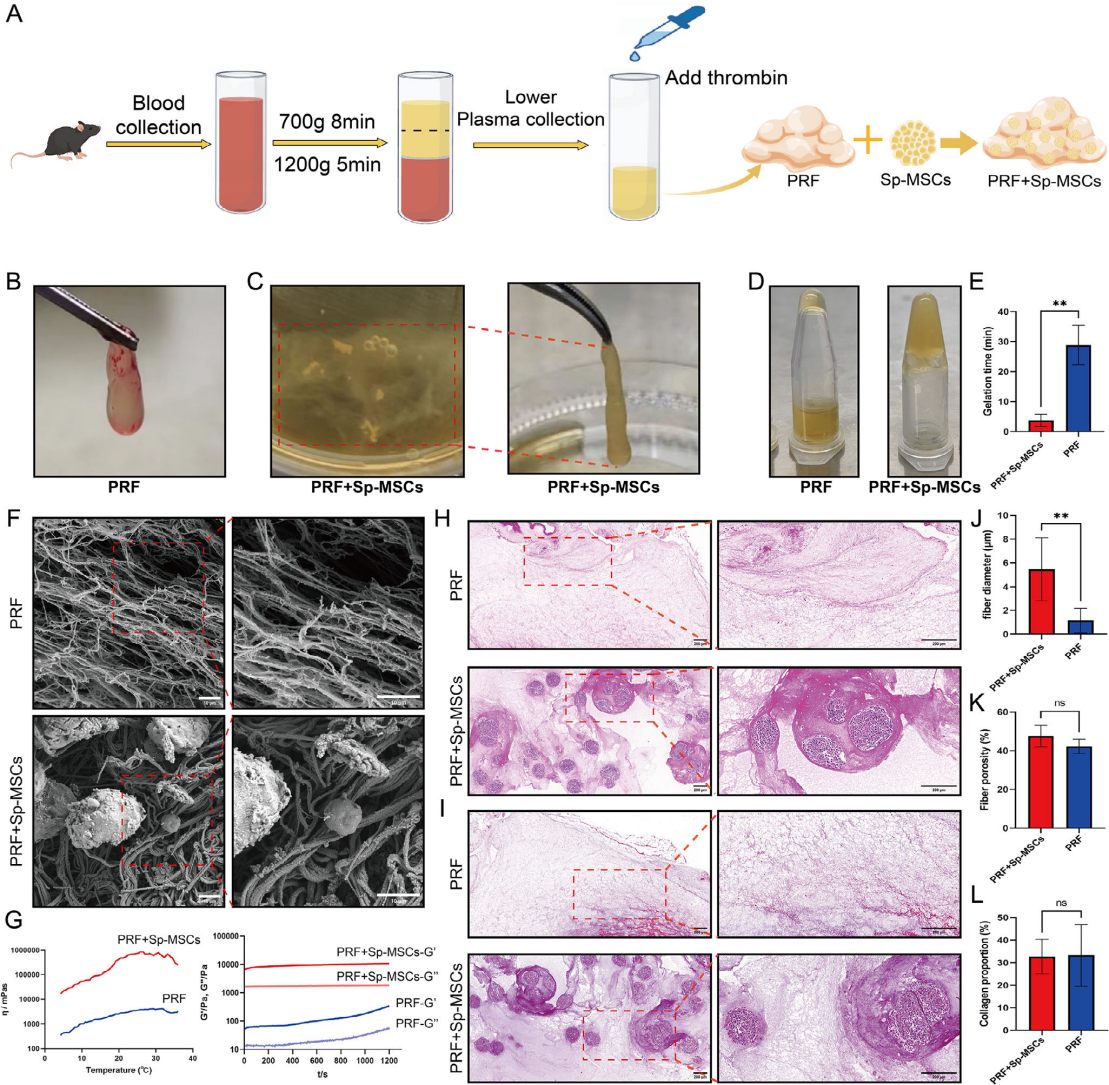
PRF combined with Sp‐MSCs enhanced PRF strength. A) Diagram of PRF preparation by differential centrifugation method and combined with Sp‐MSCs. B) Image of PRF gel. C) Cross‐linking between PRF and Sp‐MSCs. D) PRF combined with Sp‐MSCs, accelerated the coagulation and gelation of PRF. E) Quantitative analysis of gelation time of PRF and PRF+Sp‐MSCs (*n* = 3, **p < 0.01). F) Scanning electron microscope (SEM) of PRF and PRF+Sp‐MSCs. G) The addition of Sp‐MSCs enhanced the strength of PRF (Scale bar = 10 µm). G) The temperature‐viscosity curve and rheological properties of PRF and PRF+Sp‐MSCs. H,I) H&E staining, Masson staining revealed PRF‐coated Sp‐MSCs (Scale bar = 200 µm). J,K) Quantitative analysis based on SEM image of fiber diameter and porosity rate of fibers of PRF and PRF+Sp‐MSCs (*n* = 3, **p < 0.01, ns: *p* > 0.05). L) Quantitative analysis based on Masson staining collagen proportion of PRF and PRF + Sp‐MSCs (*n* = 3, ns: *p* > 0.05).

**Figure 3:**
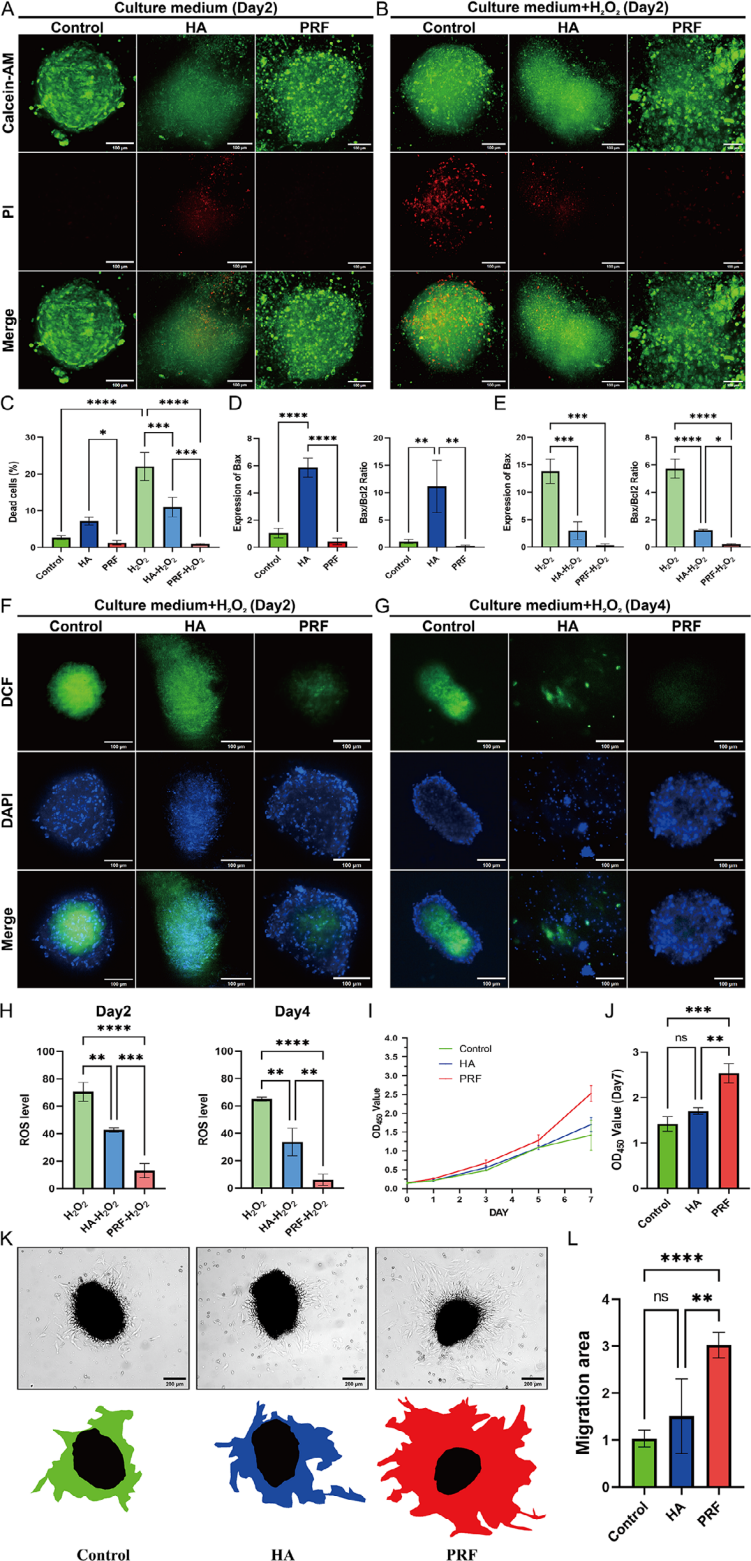
The protective efficacy of PRF for the encapsulated Sp‐MSCs. A) Live/Dead staining showing the cell viability of exposed Sp‐MSCs and encapsulated Sp‐MSCs in HA/PRF at 2 days during in vitro culture (Scale bar = 100 µm). B) Live/Dead staining showing the cell viability of exposed Sp‐MSCs and encapsulated Sp‐MSCs in HA/PRF at 2 days during in vitro culture after treatment with 500 µm H_2_O_2_ (Scale bar = 100 µm). C) Quantitative analysis based on live/dead staining of the cell viability (*n* = 3, *p < 0.05, **p < 0.01, ***p < 0.001, ****p < 0.0001). D,E) The gene expression of Bax and the ratio of Bax/Bcl2 (*n* = 3, *p < 0.05, **p < 0.01, ***p < 0.001, ****p < 0.0001, ns: *p* > 0.05). F,G) Representative fluorescent images of total intracellular ROS (stained by DCFH‐DA) in the exposed Sp‐MSCs and encapsulated Sp‐MSCs in HA/PRF at 2 and 4 days during in vitro culture after treatment with 500 µm H_2_O_2_ (Scale bar = 100 µm). H) Quantitative analysis based on DCFH‐DA staining of 2 and 4 days ROS level (*n* = 3, **p < 0.01, ***p < 0.001, ****p < 0.0001). I,J) PRF promoted proliferation of MSCs in 2D culture conditions, measured by CCK‐8 assay (*n* = 3, **p < 0.01, ***p < 0.001). K,L) PRF promoted Sp‐MSCs recovery and crawling out (*n* = 3, **p < 0.01, ****p < 0.0001).

PRF was prepared by differential centrifugation method. Sp‐MSCs were then combined directly with PRF (**Figure** **2**A,B; Figure S2A, Supporting Information). After coating Sp‐MSCs with PRF, PRF crosslinked with Sp‐MSCs (Figure 2C; Figure S2B, Supporting Information), and the gelation time was significantly shortened (Figure 2D,E). The addition of Sp‐MSCs activated the exogenous coagulation system, which made PRF coagulate into gel faster. Early application of PRF helps maintain active factors in PRF, and the shorter gelation time makes clinical application more convenient. Viscosity measurements revealed that PRF exhibited temperature‐sensitive properties. The addition of Sp‐MSCs shortened the gelation time of PRF and increased the viscosity of PRF while maintaining its temperature‐sensitive properties. Increasing temperature from 4.0 to 37.0 °C, the viscosity of PRF gel increased from 347.366 to 4194.887 η mPas^−1^, while adding Sp‐MSCs into PRF, the viscosity increased from 16 810.05 to 786 936.4 η mPas^−1^ (Figure 2G). The stimulation of the coagulation system promotes adhesion. High adherence promotes Sp‐MSC colonization in the wound site and prevents Sp‐MSCs loss due to local administration. PRF and PRF+Sp‐MSCs both exhibited hydrogel‐like behavior with elastic modulus (Gʹ) consistently surpassing the loss modulus (Gʹʹ). And the addition of Sp‐MSCs significantly enhanced the mechanical strength of PRF (Figure 2G). The result of scanning electron microscopy (SEM) showed that PRF contained a large number of fibrin components that crosslinked into a network, and Sp‐MSCs adhered to fibers (Figure 2F). After coating Sp‐MSCs, the fibers in PRF aggregated into bundles. And the fiber bundle diameter thickened from 1.15 ± 1.02 µm to 5.46 ± 2.43 µm by measuring the diameter of PRF fibrin through SEM images (Figure 3J), which revealed that the addition of Sp‐MSCs enhanced mechanical strength of PRF. The H&E and Masson staining results showed that Sp‐MSCs were disseminated in PRF fibers. And due to the activation of PRF coagulation by Sp‐MSCs, fibrin in PRF aggregated around Sp‐MSCs, forming a fibrin protective shield. Activated coagulation enriched fibrin around Sp‐MSCs without disrupting the structure of Sp‐MSCs (Figure 2H,I; Figure S2B, Supporting Information). While there was no significant change in the porosity or collagen density of PRF fibers after coating Sp‐MSCs (Figure 2K,L). PRF contained various growth factors, including epidermal growth factor (EGF) was 3.58 ± 0.79 ng mL^−1^, platelet‐derived growth factor (PDGF) was 5.19 ± 0.33 ng mL^−1^, vascular endothelial growth factor (VEGF) was 1.43 ± 0.19 ng mL^−1^, and transforming growth factor β (TGF‐β) was 6.84 ± 3.08 ng mL^−1^ (Figure S2C, Supporting Information).

### 2.3 PRF‐Shelter‐Protected Sp‐MSCs in Oxidative Stress Environment

Transplanted allogenic stem cells for diabetic wound healing are frequently subjected to oxidative stress and excessive inflammatory factors, which could cause irreparable damage to the transplanted cells and endanger therapeutic efficacy.^[^ ^17^ ^]^

Therefore, to verify the protective properties of PRF. We first cultured Sp‐MSCs in medium containing severe oxidative stress (500µM H_2_O_2_)^[^ ^27^, ^28^ ^]^ to simulate the ROS microenvironment in vitro. After 2 days and 4 days, the viability of Sp‐MSCs was assessed using the live/dead assay. The oxidative stress level was assessed using DCFH‐DA to determine the intracellular ROS levels of Sp‐MSCs. To compare the protective effects of various scaffolding materials. We selected hyaluronic acid (HA) as a typical scaffold carrier. And compared the cellular activity and intracellular ROS levels of Sp‐MSCs exposed and loaded in HA/PRF. Sp‐MSCs coated with PRF had better activity and less ROS. Under general storage conditions, Sp‐MSCs coated with HA exhibited more dead cells in the spheroids, while exposed Sp‐MSCs and Sp‐MSCs coated with PRF showed fewer dead cells. Less nutrients in HA lead to significant death of MSCs in spheroid (**Figure** **3**A,C). At the same time, the expression of apoptotic gene Bax was significantly increased in Sp‐MSCs coated with HA, and the Bax/Bcl2 ratio was also significantly increased compared to the control group and Sp‐MSCs coated with PRF (Figure 3D). Under oxidative stress conditions, exposed Sp‐MSCs caused massive dead cells in spheroids. Sp‐MSCs coated with gels showed no significant increase in dead cells. HA and PRF protected Sp‐MSCs from oxidative stress environmental interference. While Sp‐MSCs coated with HA still exhibited cell death in the spheroids (Figure 3B,C). And exposed Sp‐MSCs showed higher expression levels of Bax and Bax/Bcl2 ratio than the other group (Figure 3E). And after 4 days of storage, the shape of Sp‐MSCs coated with HA was difficult to maintain, the sphere was broken, MSCs dispersed in HA, and a large number of MSCs apoptosis occurred. The exposed Sp‐MSCs appeared a small amount of dead cells. PRF reduced the apoptosis of Sp‐MSCs while maintaining their shape (Figure S3A,C, Supporting Information). While under oxidative stress conditions, PRF continued its excellent protective ability (Figure S3B,C, Supporting Information). In addition, DCF was quantitatively analyzed for the assessment of intracellular ROS levels. The results showed that in the oxidative stress environment, exposed Sp‐MSCs consistently maintained high levels of ROS. And ROS levels decreased when coated with HA and PRF. Although HA can provide a physical barrier to assist Sp‐MSCs in resisting adverse environments. The lack of nutrients in HA leads to broken sphere and extensive cell apoptosis of Sp‐MSCs. Meanwhile, Sp‐MSCs coated with PRF showed the lowest levels of ROS in spheroids (Figure 3F–H). The above results indicated that PRF shielded Sp‐MSCs by enriching fibrin around Sp‐MSCs, protect from adverse conditions while providing nutrients for maintaining activity of Sp‐MSCs had the lowest cell death rate and ROS levels in spheroids (Figure 3A–H; Figure S3A–C, Supporting Information).

Before re‐plating Sp‐MSCs onto the culture plate, the culture plate was wrapped with HA/PRF. Sp‐MSCs were re‐plated onto culture dishes and then covered with HA/PRF. After 24 h, the image showed that PRF significantly promoted the recovery migration of Sp‐MSCs, accelerating the speed of MSCs crawling out and migrating from the spheroids, but HA had no significant promoting effect (Figure 3K,L). The fibrin scaffold in PRF provided an effective scaffold for Sp‐MSCs recovery and climbing. Growth factors in PRF also promoted MSC migration, and the mechanical strength of the PRF gel was closer to the tissue. These variables allowed Sp‐MSCs to remain active and recover more quickly in PRF. The CCK‐8 analysis showed that PRF significantly promoted MSC viability (Figure 3I,J). Under 2D culture conditions, HA had no significant effect on individual MSC activity, proving that HA itself had no significant toxicity in 2D cell culture. Under 3D conditions, HA‐coated Sp‐MSCs, reducing the nutrients obtained inside Sp‐MSCs. The above results indicated that PRF could protect Sp‐MSCs from oxidative stress conditions, while maintaining Sp‐MSCs activity and promoting MSC proliferation.

### 2.4 PRF‐Regulated Sp‐MSCs Secretion Activity and Foster Microenvironment for Angiogenesis

**Figure 4:**
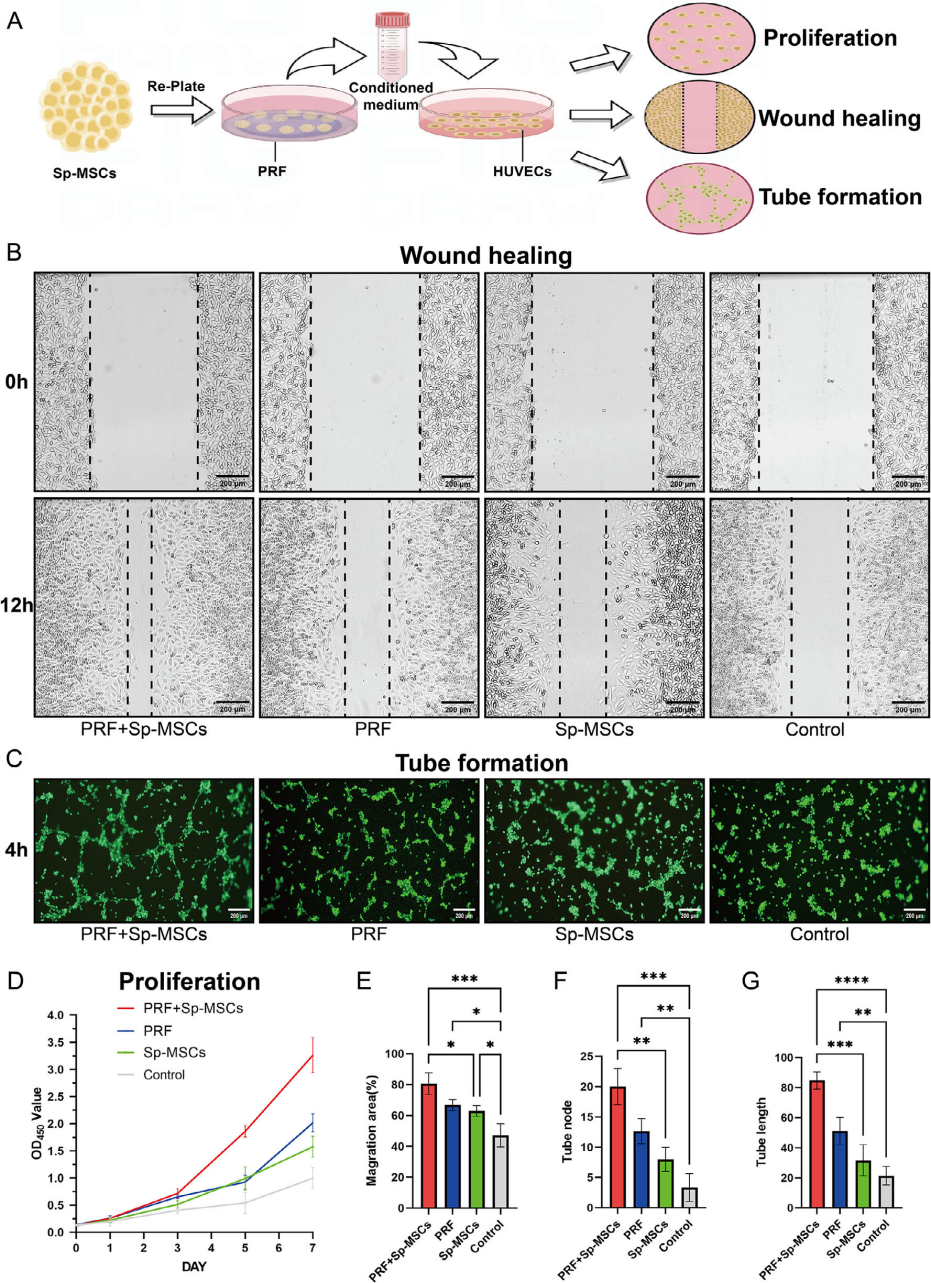
Effect of PRF combined with Sp‐MSCs on angiogenesis in vitro. A) Schematic diagram of exploring the effect of PRF treated conditioned medium (CM) on proliferation, migration, and tube formation of HUVECs. B) PRF combined with Sp‐MSCs enhanced HUVECs migration (Scale bar = 200 µm). C) PRF combined with Sp‐MSCs created a microenvironment that promoted angiogenesis (Scale bar = 200 µm). D) PRF combined with Sp‐MSCs enhanced HUVECs proliferation (*n* = 3). E) Healing rate of HUVECs in the scratch assay (*n* = 3, *p < 0.05, **p < 0.01, ***p < 0.001). F,G) Quantitative analysis of tube node and length of tube formation results (*n* = 3, **p < 0.01, ***p < 0.001, ****p < 0.0001).

Diabetic wounds are difficult to heal due to the reduction of new blood vessels in the wound site, which causes slow local tissue regeneration.^[^ ^29^ ^]^ Therefore, the formation of an angiogenesis‐promoting microenvironment in the wound site is critical for diabetic wound healing.^[^ ^30^ ^]^ To verify the effect of PRF combined with Sp‐MSCs on angiogenesis, Sp‐MSCs were replated on PRF. And the condition culture medium (CM) of Sp‐MSCs was collected and used to culture human umbilical vein endothelial cells (HUVECs). Then assessed proliferation, migration, and tube formation ability of HUVECs (**Figure** **4**A). The CCK‐8 assay showed that PRF+Sp‐MSCs significantly promoted HUVEC proliferation activity. Compared to the control group, PRF group and Sp‐MSCs group also had a promoting effect (Figure 4D). The migration ability of HUVECs was assessed using the scratching assay. PRF+Sp‐MSCs group showed a higher wound healing rate than the other groups. PRF group and Sp‐MSCs group were higher than control group (Figure 4B,E). In vitro tube formation capacity was evaluated and the PRF+Sp‐MSCs group showed a higher number of branches and the longest total branch length. The PRF group promoted angiogenesis more effectively than the Sp‐MSCs group due to the rich VEGF content in PRF (Figure 4C,F,G). These results collectively demonstrated the capacity of PRF combined with Sp‐MSCs to promote HUVECs migration and angiogenesis, creating an angiogenesis‐promoting microenvironment. By measuring the growth factors secreted by Sp‐MSCs after recovery. We found that compared to Sp‐MSCs, Sp‐MSCs treated with PRF showed a certain enhancement in their ability to secrete EGF, VEGF, and TGF‐β (Figure S3D, Supporting Information). PRF accelerated the recovery of Sp‐MSCs, enhanced their activity, and promoted their secretion function. Together, they formed a microenvironment that was suitable for local tissue repair of wounds, especially vascular regeneration.

### 2.5 Exploration of the Transcriptomic Profile of PRF‐Coated Sp‐MSCs

The data presented above indicated PRF's protective efficacy in shielding Sp‐MSCs from oxidative stress while also promoting angiogenesis. Thus, to exploit the underlying mechanism of how PRF exerts protective functions and enhances the repair functions of Sp‐MSCs. RNA‐seq was used to analyze the transcriptomic changes of the Sp‐MSCs with and without PRF.

**Figure 5:**
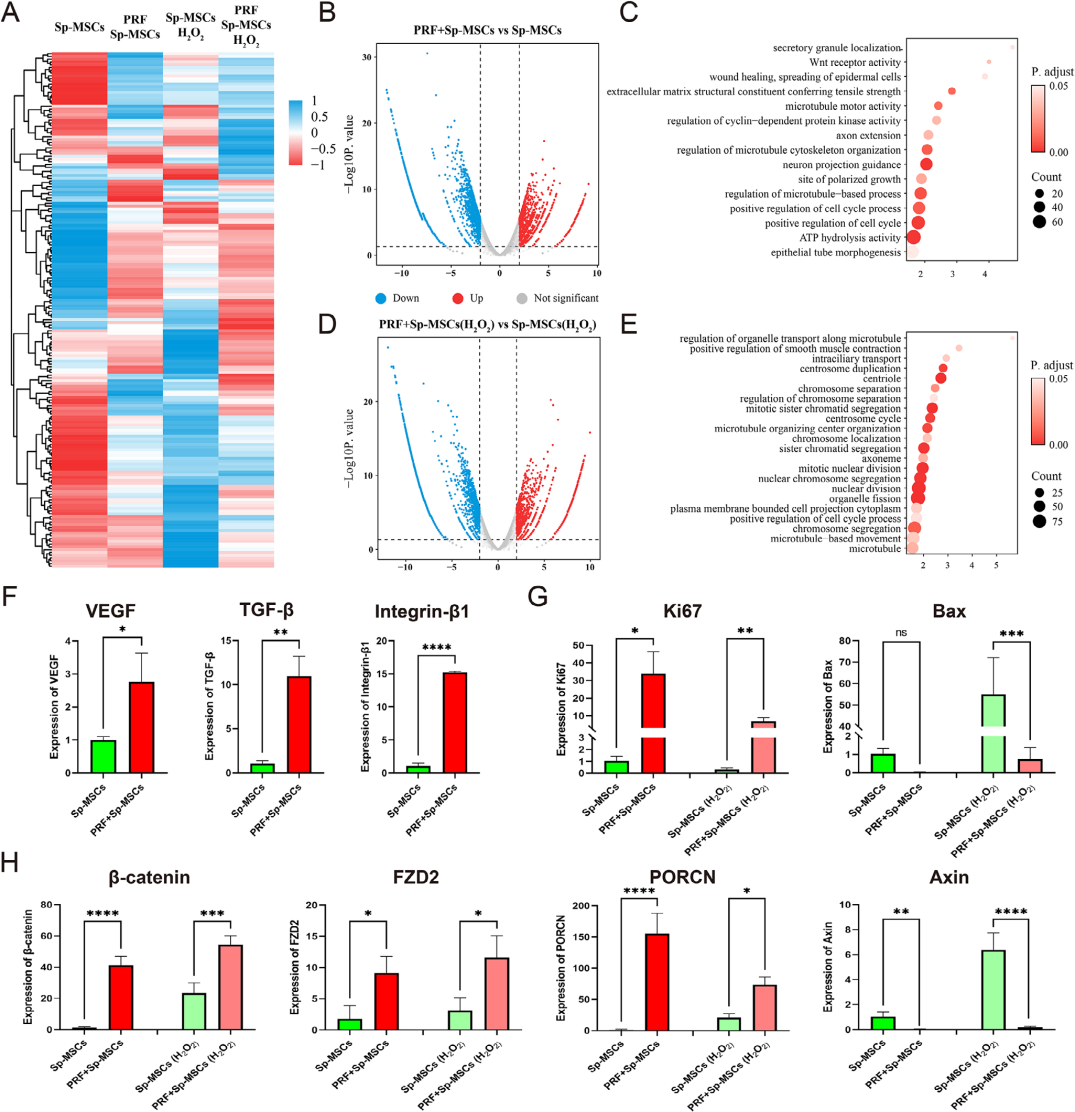
Exploration of transcriptome profile patterns and functional enrichment analysis. A) The heatmap transcriptomic profiling of Sp‐MSCs, PRF‐coated Sp‐MSCs, and Sp‐MSCs (H_2_O_2_), PRF‐coated Sp‐MSCs (H_2_O_2_), (H_2_O_2_ mean treatment with 500 µm H_2_O_2_). B) Volcano map of differentially expressed genes of Sp‐MSCs and PRF‐coated Sp‐MSCs. C) KEGG pathway enrichment analysis of differentially expressed genes might take place between PRF‐coated Sp‐MSCs vs Sp‐MSCs. D) Volcano map of differentially expressed genes of Sp‐MSCs and PRF‐coated Sp‐MSCs treatment with 500 µm H_2_O_2_. E) KEGG pathway enrichment analysis of differentially expressed genes might take place between PRF‐coated Sp‐MSCs vs Sp‐MSCs treatment with 500 µm H_2_O_2_. F) The expression level of VEGF, TGF‐β, and Integrin‐β1 of Sp‐MSCs and PRF‐coated Sp‐MSCs. G) The expression level of Ki67 and Bax of Sp‐MSCs, PRF‐coated Sp‐MSCs and Sp‐MSCs(H_2_O_2_), PRF‐coated Sp‐MSCs(H_2_O_2_). H) The expression level of β‐catenin, FZD2, PORCN, and Axin of Sp‐MSCs, PRF‐coated Sp‐MSCs, and Sp‐MSCs(H_2_O_2_), PRF‐coated Sp‐MSCs(H_2_O_2_). (*n* = 3, *p < 0.05, **p < 0.01, ***p < 0.001, ****p < 0.0001, ns: *p* > 0.05).

Firstly, we investigated the transcriptomic profile of Sp‐MSCs uncovered versus combined with PRF to explore the effects of PRF on intracellular responses of MSCs. Generally, there were 3625 downregulated and 1506 upregulated all differentially expressed genes (DEGs) between PRF‐coated Sp‐MSCs vs Sp‐MSCs (**Figure** **5**B). Further, Gene ontology (GO) enrichment analysis was performed for the downregulated and upregulated DEGs, respectively. Kyoto Encyclopedia of Genes and Genomes (KEGG) enrichment analysis was enriched in protein synthesis and secretion, epithelial and neural regeneration, cell cycle, and energy metabolism. After Sp‐MSCs bind to PRF, increased protein secretion function and enhanced cellular activity of MSCs (Figure 5C). KEGG enrichment analysis further exhibited significant signaling pathways and indicated that the addition of PRF enhances the biological activity and secretion function of MSCs. We validated the secretion‐related genes VEGF and TGF‐β, as well as the proliferation‐related gene Ki67. PRF‐coated Sp‐MSCs showed enhanced secretion and proliferation activity. Integrins are a family of cell adhesion receptors.^[^ ^31^ ^]^ Integrin‐β1 plays an important role in MSC migration, paracrine secretion, and angiogenesis regulation.^[^ ^32^, ^33^ ^]^ PRF‐coated Sp‐MSCs showed a significant increase in integrin‐β1 and VEGF expression (Figure 5F). Therefore, activation of the Integrity pathway may play a key role in the enhanced repair function of PRF‐coated Sp‐MSCs.

To further explore the protective effect of PRF under an inflammatory microenvironment, we cultured PRF‐coated Sp‐MSCs vs exposed Sp‐MSCs in a ROS‐rich environment in vitro and analyzed the change of the transcriptome in MSCs. It was found that there were 4001 downregulated and 1325 upregulated DEGs between the two groups (Figure 5D). KEGG enrichment analysis was enriched in cell cycle and cell division‐related functions (Figure 5E), indicated that PRF protected Sp‐MSCs in an oxidative stress environment and maintained MSC proliferation activity. In the oxidative stress environment, PRF‐coated Sp‐MSCs maintained high expression levels of Ki67 while reducing the expression of apoptosis‐related gene Bax (Figure 5G). According to KEGG enrichment results (Figure 5C,E) PRF‐coated will activate Wnt‐related pathway in Sp‐MSCs. Wnt/β‐catenin signaling plays a crucial role in the migration of MSCs, apoptosis inhibition.^[^ ^34^, ^35^ ^]^ Meanwhile, activation of Wnt/β‐catenin pathway promotes the proliferation of MSCs under both 2D and 3D conditions.^[^ ^36^ ^]^ PRF‐coated Sp‐MSCs showed a significant increase of Wnt/β‐catenin pathway‐related genes β‐catenin, Frizzled class receptor (FZD2) and Porcupine homolog (PORCN), simultaneously reducing the expression of Axis inhibitor (Axin), a negative regulator of Wnt/β‐catenin pathway (Figure 5H). Therefore, PRF not only uses a fibrin network to coat Sp‐MSCs to protect from adverse environment, simultaneously activated Wnt/β‐catenin pathway, enhances the survival ability of Sp‐MSCs in adverse environments, and reduced MSCs apoptosis.

### 2.6 PRF Combined with Sp‐MSCs Promoted Diabetic Wound Healing

**Figure 6:**
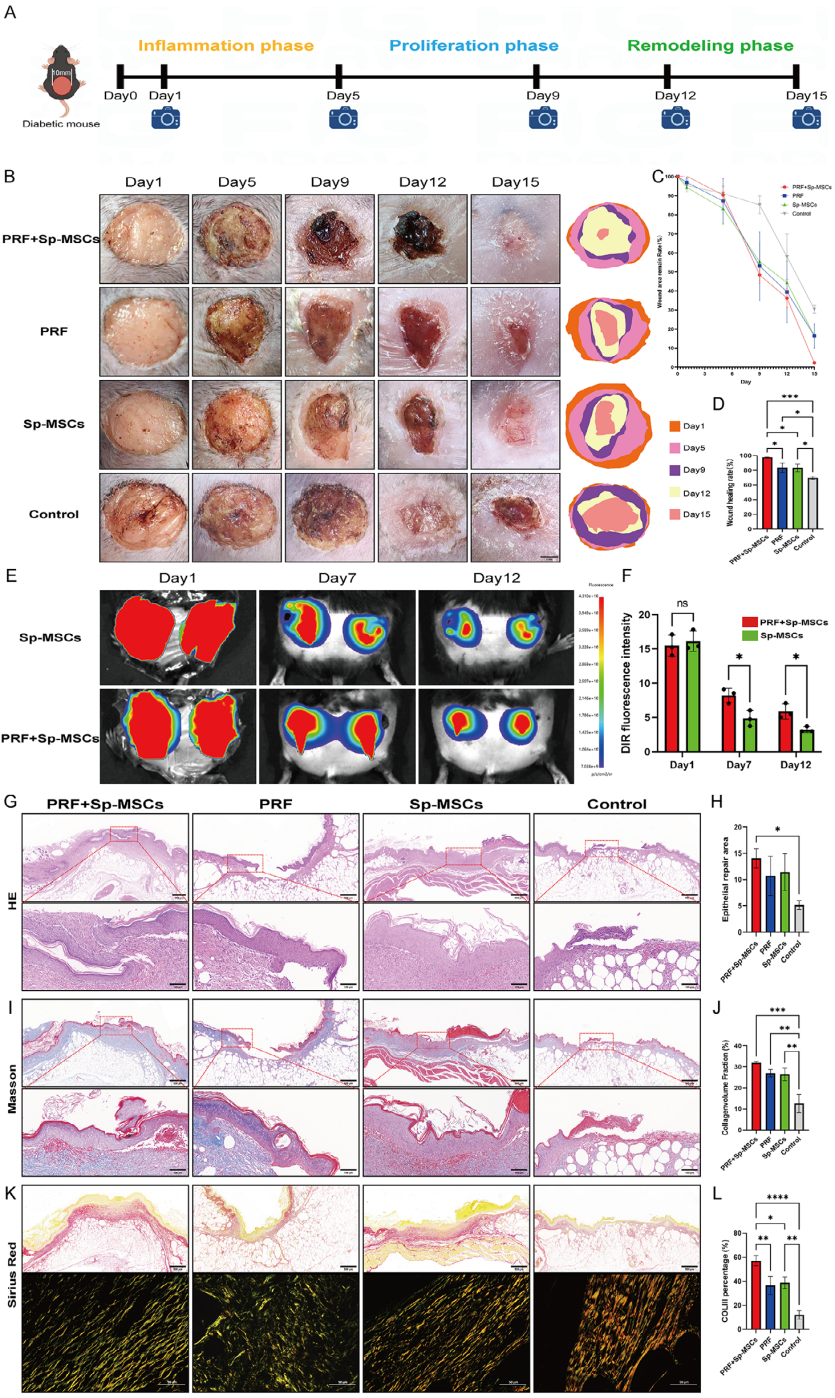
Effects of PRF combined with Sp‐MSCs in promoting diabetic wound healing in db/db mouse. A) Schematic diagram of establishing a diabetic wound and the timeline of animal experiments to test the therapeutic effect of PRF combined with Sp‐MSCs. B) Representative images of the wound at days 1, 5, 9, 12, and 15 and the diagrams of time‐evolved wound areas (Scale bar = 2 mm). C) Statistical analysis line chart of the wound area. D) Statistical analysis of wound healing rate (*n* = 3, *p < 0.05, ***p < 0.001). E) The engraftment of DiR‐labeled Sp‐MSCs detected by ex vivo imaging at different time points post‐transplantation. F) Quantification of the fluorescent signal intensity of the transplanted cells at different time points (*n* = 3, *p < 0.05, ns: p>0.05). G,H) H&E staining of wound tissues at day 15, and quantification of the epidermis repair area of wounds at day 15 (Scale bar = 100/500 µm) (*n* = 3, *p < 0.05). I,J) Masson staining of wound tissues at day 15, and quantification of the epidermis repair area of wounds at day 15 (Scale bar = 100/500 µm) (*n* = 3, **p < 0.01, ***p < 0.001). K,L) Sirius red staining of wound tissues at day 15, and quantification of type III collagen percentage of wounds at day 15 (Scale bar = 50/500 µm) (*n* = 3, *p < 0.05, **p < 0.01, ****p < 0.0001).

In this study, we used a gene mutation mouse model (db/db mouse) to establish a diabetic wound model. Subsequently, the diabetic mice were allocated into four groups (Control/Sp‐MSCs/PRF/PRF+Sp‐MSCs) (**Figure** **6**A). To vividly show the process of wound healing, photos were taken on days 1, 5, 9, 12, and 15 to record the changes in the wound area (Figure 6B). Statistical results showed that among all groups (Figure 6C), the PRF+Sp‐MSCs group had the highest wound healing rate (97.7±0.38%) on day 15. The PRF group and Sp‐MSCs group had lower healing rates (83.5% ± 5.18% and 83.4% ± 4.28%), while the healing rate of the control group was only 69.6% ± 1.68% (Figure 6D).

To better investigate the therapeutic effect of PRF combined with Sp‐MSCs, transplanted Sp‐MSCs were labeled with a far‐red plasma membrane fluorescent probe and then seen applying ex vivo imaging analysis. Notably, Sp‐MSCs coated with PRF exhibited a higher cell viability. The fluorescent signals from the PRF‐coated Sp‐MSCs were higher than Sp‐MSCs. Indicating the in vivo cytoprotective effect of PRF, which protects Sp‐MSCs from the complex immune microenvironment (Figure 6E,F).

To explore the effect of PRF combined with Sp‐MSCs on the immune regulation of the inflammation phase and regeneration of the proliferation phase during diabetic wound healing. H&E staining were applied to skin specimens on day 5 to observe the overall condition of wound healing. PRF+Sp‐MSCs group had migrated epithelial tissue around the wound site. While there was no regenerated epithelium found at the wound site of the other three groups (Figure S4A, Supporting Information). It indicated that PRF combined with Sp‐MSCs enabled diabetic wounds to enter the proliferation phase earlier. In the early stage of diabetic wound healing, the reconstruction of vascular regeneration microenvironment plays a vital role in accelerating wound healing.^[^ ^37^ ^]^ Further, immunofluorescence staining of CD34, α‐smooth muscle actin (αSMA), and VEGF showed PRF combined with Sp‐MSCs created a positive microenvironment for angiogenesis for diabetic wound healing (Figure S4B,D, Supporting Information). More VEGF and vascular regeneration cells will make revascularization and faster tissue repair during the proliferation and remodeling phase. Meanwhile, PRF combined with Sp‐MSCs jointly regulated the inflammation phase. PRF+Sp‐MSCs reduced neutrophil recruitment and the expression of pro‐inflammatory cytokines like tumor necrosis factor‐alpha (TNF‐α), and promoted the macrophage polarization towards M2 phenotype (Figure S4C,E, Supporting Information). PRF combined with Sp‐MSCs effectively regulates the complex immune microenvironment of diabetic wounds, and shortens the inflammatory phase.

Then we performed histopathological analysis on day 15. H&E and Masson staining were applied to skin specimens to observe the collagen fibers and appendage formation in the wound. The newly generated epithelial tissue in the PRF+Sp‐MSCs group had a larger repair area (Figure 6G,H) and a higher proportion of new collagen coverage (Figure 6I,J). In the newly formed collagen of the skin. Type III collagen is more expressed in the dermis of newly created skin collagen, providing the skin with flexibility. And type I collagen is densely arranged and has a relatively hard texture.^[^ ^38^ ^]^ Sirius red staining was utilized to investigate type I and III collagen on the border of the wound, and the result showed that the PRF+Sp‐MSCs group showed a larger proportion of type III collagen. The newborn collagen of the control group was mainly type I. The PRF+Sp‐MSCs group exhibited more collagen regeneration and a collagen ratio closer to normal skin (Figure 6K,L).

### 2.7 PRF and Sp‐MSCs Regulated the Inflammatory Environment of Diabetic Wounds and Promoted Tissue Repair

**Figure 7:**
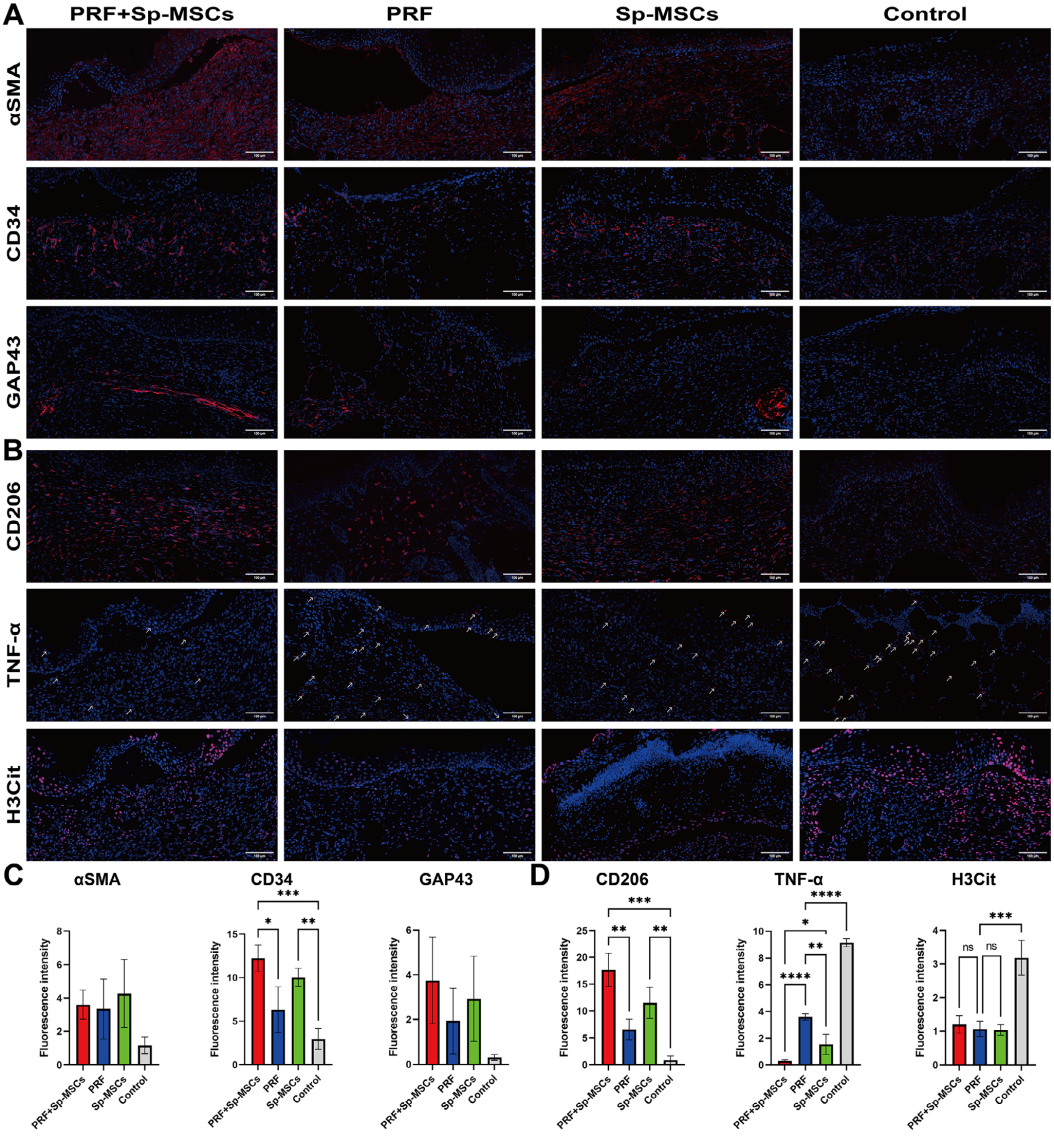
The effect of PRF combined with Sp‐MSCs on tissue regeneration and immune regulation of diabetic wound healing. A) Immunofluorescence of αSMA and CD34 immunostaining showed accumulation neovascularization, and GAP43 immunostaining showed neuroregeneration at the wound on day 15 (Scale bar = 100 µm). B) Immunofluorescence of TNF‐α (white arrow) immunostaining showed accumulation of pro‐inflammatory factors, CD206 immunostaining showed accumulation of M2 macrophages, and H3Cit immunostaining showed neutrophil recruitment at the wound on day 15 (Scale bar = 100 µm). C,D) Quantitative analysis based on immunostaining of αSMA, CD34, GAP43, CD206, TNF‐α, H3Cit (*n* = 3, *p < 0.05, **p < 0.01, ***p < 0.001, ****p < 0.0001, ns: *p* > 0.05).

Tissue immunofluorescence staining was performed to investigate regeneration and immunological regulation in the wound site of day 15. Immunofluorescence staining of CD34 and αSMA showed that PRF+Sp‐MSCs group and Sp‐MSCs group promoted angiogenesis in vivo (**Figure** **7**A,C). Growth‐associated protein‐43 (GAP43) staining was used to observe nerve tissue regeneration, and both PRF+Sp‐MSCs group and Sp‐MSCs group showed more nerve tissue in the wound site (Figure 7A,C).

Immunofluorescence results indicated that PRF+Sp‐MSCs group and Sp‐MSCs group had significantly higher rates of M2 type macrophages (CD206) and reduced inflammatory factors such as TNFα in the wound site (Figure 7B,D). While the immune regulatory ability of the PRF group was better than that of the control group. But fell short of PRF+Sp‐MSCs group and Sp‐MSCs group. It was indicated that Sp‐MSCs played a role in regulating immunity and accelerating the transformation of wounds into anti‐inflammatory phenotypes. PRF+Sp‐MSCs, PRF, and Sp‐MSCs group reduced neutrophil recruitment than the control group. (Figure 7B,D). These results collectively demonstrated that PRF combined with Sp‐MSCs could regulate the immune environment in the wound site while also promoting tissue repair and regeneration.

## 3 Discussion

Diabetic wounds are among the most severe complications of chronic diabetes, and stem cell therapy has emerged as a promising treatment approach.^[^ ^39^, ^40^, ^41^ ^]^ However, precisely delivering mesenchymal stem cells (MSCs) to the wound site and maintaining their activity remain challenging.^[^ ^42^ ^]^ The local microenvironment in diabetic wounds is characterized by the aggregation of inflammatory cells, an increase in pro‐inflammatory factors, and a deficiency in blood supply and nourishment. It is difficult for exogenous therapeutic stem cell drugs to remain active in the diabetic microenvironment, which significantly reduces their reparative effects. To address this issue, our study employed PRF gel, derived from autologous plasma, to coat Sp‐MSCs for the treatment of diabetic wounds.

### 3.1 Sp‐MSCs Accelerated PRF Coagulation and Enhanced Mechanical Strength of PRF

Sp‐MSCs were prepared using an optimized method involving the formation of spheroids containing 5000 MSCs each, and we have previously confirmed their excellent performance in vitro and in vivo.^[^ ^12^ ^]^ In this study, we verified these findings (Figure 2).

Then we prepared PRF gel from autologous plasma by differential centrifugation and coated Sp‐MSCs with PRF (Figure 2A). PRF contains various bioactive compounds from platelets consisting of chemokines, growth factors, and cytokines (Figure S2C, Supporting Information). These could activate angiogenesis, alter extracellular matrix (ECM) remodeling, and promote cell recruitment and proliferation,^[^ ^26^ ^]^ as well as act against bacterium infections.^[^ ^43^ ^]^ Furthermore, PRF is entirely derived from the autologous plasma of patients, has high biocompatibility, and produces no immune rejection reaction. We coated Sp‐MSCs with PRF (Figure 2). The addition of Sp‐MSCs shortened the gelation time of PRF and enhanced the adhesion and mechanical strength of PRF (Figure 2D,E). Improved the shortcomings of prolonged coagulation time and lower PRF mechanical strength (Figure 2G). It is conducive to accurate delivery of Sp‐MSCs at the wound site. Upon interaction with PRF, Sp‐MSCs trigger the coagulation cascade of PRF, subsequently augmenting its mechanical strength. (Figure 2D–L).

### 3.2 PRF Shielding Sp‐MSCs Against ROS and Noxious Inflammatory Factors

In the treatment of diabetic wounds, Sp‐MSCs necessitate a carrier capable of precisely targeting the wound site, safeguarding the cells from the detrimental ROS environment, and preserving their viability. PRF serves as a scaffold for Sp‐MSCs, more importantly, with its intricate network of cross‐linked fibrin offering a robust shield for their growth (Figure 2H,I). Fibrin in PRF exhibits a significant capacity to counteract ROS (Figure 3B–H). Moreover, PRF is enriched with growth factors and plasma components that are essential for sustaining and enhancing the functional activity of Sp‐MSCs (Figures S2C and S3A, Supporting Information). The application of PRF to diabetic wounds has been observed to modulate the immunological microenvironment, thereby creating a protective shield for Sp‐MSCs in adverse environment.^[^ ^43^, ^44^ ^]^ Furthermore, PRF has demonstrated the ability to expedite the migratory recovery and extrusion of Sp‐MSCs (Figure 3K,L), thereby enabling a swift initiation of tissue reparative processes. Sp‐MSCs coated with PRF have been shown to withstand the intricacies of the diabetic wound microenvironment, protect the cells from ROS, and consequently augmenting their survival duration and rate within the wound (Figure 6E,F). Transcriptomic analysis has revealed that, PRF can activate related genes of Integrin‐β1‐VEGF pathway of MSCs, thereby promoting MSCs migration, proliferation, and secretion, enhancing their tissue repair function (Figure 5B,C,F). At the same time, under oxidative stress conditions, PRF can activate the related genes to Wnt/β‐catenin pathway to protect MSCs activity and reduce apoptosis under adverse conditions (Figure 5B–E,G,H). In the complex microenvironment of diabetic wounds, PRF provides physical protection for Sp‐MSCs, mitigating the impact of adverse environment, and concurrently orchestrates a transition towards an anti‐inflammatory state by immune regulation (Figure 7B; Figure S4C, Supporting Information).^[^ ^43^, ^44^ ^]^ PRF serves as an adept shield for Sp‐MSCs, safeguarding them against ROS and noxious inflammatory factors.

### 3.3 PRF Enhanced Repair Functions of Sp‐MSCs

PRF serves as a protective shield and nurturing environment for Sp‐MSCs. The growth factors and plasma components within PRF, particularly the alpha‐granules, create a conducive nutritional milieu for Sp‐MSCs.^[^ ^45^ ^]^ Sp‐MSCs' recovery migration was accelerated (Figure 3K,L), and their proliferation activity was increased (Figure 3I,J). We believe this is closely related to the presence of a multitude of reparative factors in PRF, For instance, platelet‐derived growth factors A and B (PDGF‐A and B), transforming growth factor β (TGF‐β), and vascular endothelial growth factor (VEGF) (Figure S2C, Supporting Information).^[^ ^46^, ^47^ ^]^ The growth factors released from PRF could effectively enhance Sp‐MSCs repair function. PRF could not only accelerate the recovery and migration of Sp‐MSCs and enhance the proliferative activity of MSCs, but also enhance MSC paracrine function ability to repair and regenerate tissues (Figure 4). The reduction of neovascularization, which leads to insufficient local blood supply is the main factor of difficult wound healing in diabetes.^[^ ^48^ ^]^ The growth factors released from PRF could effectively promote vascular regeneration and tissue repair (Figure S3D, Supporting Information).^[^ ^49^ ^]^ Meanwhile, PRF enhanced the metabolic activity and secretion function of Sp‐MSCs. It has been found that the combination of PRF with Sp‐MSCs could establish a microenvironment that fosters vascular regeneration and facilitates tissue repair.^[^ ^50^, ^51^, ^52^, ^53^ ^]^ Sp‐MSCs coated with PRF gel enhanced the proliferation and migration ability of surrounding cells through secretion (Figure 4B,D,E). PRF‐coated Sp‐MSCs also formed a favorable microenvironment for angiogenesis (Figure 4C,F,G). It has been indicated that PRF protects Sp‐MSCs from adverse environmental assaults, internally fortifying their reparative functions, which include the enhancement of recovery migration, proliferation, and secretion of Sp‐MSCs (Figures 3 and 4; Figure S3, Supporting Information).

In vivo, Sp‐MSCs encapsulated with PRF significantly promoted wound healing in diabetes. This synergistic combination has been shown to foster the regeneration of skin, blood vessels, and nerves within diabetic wounds (Figures 6 and 7; Figure S4, Supporting Information). More importantly, Sp‐MSCs encapsulated with PRF improved the inflammatory environment of diabetic wounds. Macrophages are innate immune cells with important roles in immunoregulation and tissue remodeling.^[^ ^54^, ^55^ ^]^ Sp‐MSCs encapsulated with PRF transformed macrophages into M2 phenotype in the wound site and reduced inflammatory factors. At the same time, the aggregation and dysfunction of neutrophils in the wound site also indicate that diabetic wounds are prone to infection and immune dysfunction.^[^ ^56^, ^57^ ^]^ PRF and Sp‐MSCs jointly regulated the immune microenvironment in the wound, reducing the release of inflammatory factors and the recruitment of neutrophils (Figure 7B; Figure S4C, Supporting Information).

In summary, PRF acts as a biocompatible shield that not only protects Sp‐MSCs but also amplifies their therapeutic potential, making it a promising strategy for enhancing tissue repair and regeneration in diabetic wounds.

### 3.4 Limitations of PRF and the Emerging Potential of Biomimetic PRF Alternatives

PRF) is recognized for its superior biocompatibility and significant regenerative potential; however, the autologous plasma from which it is derived imposes certain constraints. Initially, the mechanical properties of PRF necessitate further strengthening.^[^ ^58^ ^]^ Additionally, while PRF encompasses an array of growth factors, the ideal release kinetics to facilitate the healing of diabetic wounds remain undefined.^[^ ^59^ ^]^ Moreover, the inability to mass‐produce PRF, due to the constraints of blood collection and centrifugation processes, limits its widespread application in wound care.^[^ ^23^, ^60^ ^]^

The characteristics of PRF, such as platelet concentration, fibrin structure, and growth factor content, exhibit natural variability, which may be influenced by factors including individual differences, blood collection methods, and centrifugation speed.^[^ ^24^, ^61^ ^]^ Individual differences, such as age, gender, and health status, may affect the platelet concentration and growth factor content of PRF.^[^ ^62^, ^63^ ^]^ Blood collection methods, including the site, volume, and timing of blood collection, may also lead to differences in PRF characteristics.^[^ ^64^, ^65^ ^]^ Moreover, centrifugation speed, a key factor in PRF preparation, can result in different platelet concentrations and fibrin structures in PRF,^[^ ^66^ ^]^ thereby affecting its biological properties. In this study, we found that PRF can effectively protect Sp‐MSCs from the harmful effects of ROS in diabetic wounds, thereby exerting a more sustained and beneficial effect. These findings highlights the importance of PRF characteristic variability for its long‐term clinical implications, including the effectiveness of tissue repair and regeneration, the modulation of inflammatory responses, and the development of personalized treatment strategies. Future research should focus on elucidating the mechanisms underlying PRF characteristic variability and on optimizing preparation methods and treatment protocols to maximize the therapeutic potential of PRF in tissue repair and regeneration.

Recently, there has been a surge in investigative focus on the regenerative capabilities of PRF and other plasma‐derived products. Platelets, known for their distinctive α‐granules, exhibit high regenerative potential.^[^ ^67^ ^]^ These α‐granules are repositories for a variety of growth factors, chemokines, and antimicrobial proteins, and the unique nature of these granules confers a pivotal role to platelets in tissue regeneration.^[^ ^68^ ^]^ Ongoing research into the constituent factors and underlying molecular mechanisms is progressively elucidating their roles in the enhancement of regenerative processes, coagulation, and other related functions.^[^ ^69^, ^70^, ^71^ ^]^ Concurrently, the development of innovative hemostatic agents, such as biomimetic platelets or platelet‐mimetic particles,^[^ ^72^, ^73^, ^74^ ^]^ is charting new courses for the design and engineering of biomaterials. The advent of biomimetic α‐granules or synthetic, platelet‐analogous biomaterials may surmount the challenge of mass production faced by plasma‐derived biomaterials. These advanced materials promise consistent active factor content within the gel matrix, independent of inter‐individual variability, and allow for the utilization of diverse scaffold materials tailored to specific application scenarios. Autologous plasma gel emerges as an accessible, cost‐effective, and biosafe bioactive material, closely resembling native tissue in composition and mechanical properties. The encapsulation of Sp‐MSCs within PRF not only bolsters mechanical attributes but also affords these cells external protection, internal scaffold adhesion, and nutritional support. The utilization of biomaterial gels derived from autologous plasma paves novel avenues and strategies for the future design of regenerative medicine materials.

## 4 Conclusion

In summary, our study has put forth a strategy for the precise delivery of Sp‐MSCs using PRF. Our findings further reveal that the incorporation of Sp‐MSCs can accelerate the gelation process of PRF and enhance its mechanical strength. This PRF delivery approach not only provides fibrin protection for Sp‐MSCs against the detrimental effects of Reactive Oxygen Species (ROS), but also enhances their functional capabilities, including promoting recovery migration and proliferation. Moreover, this strategy stimulates the secretory activity of Sp‐MSCs, collectively creating a microenvironment conducive to cell proliferation and angiogenesis. Ultimately, the synergistic effect of PRF and Sp‐MSCs modulates the immune microenvironment, fostering epithelial, neural, and vascular regeneration in diabetic wounds. This combination maintains the activity of Sp‐MSCs and enhances their reparative efficiency in diabetic wound healing, thereby accelerating the healing process. Therefore, our results indicate that PRF serves as an effective carrier and shield for the delivery of Sp‐MSCs in the treatment of diabetic wounds, and the development of smart gel materials that mimic PRF is an extremely promising direction for future research.

## 5 Experimental Section

### E‐MSCs Culture and Spheroidal Formation

E‐MSCs were cultured on gelatin (STEMCELL Technologies #07903) in MSC culture medium prepared with αMEM (Gibco #12571) and 10% HPL (Compass Biomedical #PLS‐6), 1% GlutaMAX (Gibco #35050), and 1% NEAA (Gibco #11140). And E‐MSCs were generated as described previously.^[^ ^10^, ^75^, ^76^ ^]^ The cells were cultured at 37 °C in the presence of 5% CO_2_ with a medium change every 3 days.^[^ ^10^, ^12^ ^]^

MSCs were digested and cell suspension was prepared. Anti‐adhesion (STEMCELL Technologies #0 7010) treatment Agreewell800 (STEMCELL Technologies #34 811) was used. Cell suspension was added to each well, after centrifugation (100 × *g*, 3 min), cultured for 24 h to form Sp‐MSCs. Then Sp‐MSCs were observed and collected in 15 mL centrifuge tubes, MSC culture medium was added, and stored at room temperature (25 °C).

Cell suspensions of different concentrations were added to form cell spheroids in Agreewell800 (STEMCELL Technologies #34 811) of varying sizes.^[^ ^12^ ^]^ The number of Sp‐MSCs in the sphere‐forming plate was counted under an inverted microscope. And photos were taken to measure the diameter of Sp‐MSCs. After collecting Sp‐MSCs, Sp‐MSCs were left to settle, the supernatant of the culture medium was taken, and the number of free cells was counted by cell counting.

(1) $$Sphere\;formation\;rate=\frac{Sp-MSCs\;formed\;in\;perwell}{Total\;number\;of\;micropores\;perwell}$$

(2) $$Free\;cells\;rate=\frac{The\;amount\;of\;free\;cells}{Total\;amount\;of\;added\;cells}$$

### Live/Dead Cell Assay

Sp‐MSCs were stored in MSC complete culture medium and incubated at 25 °C for 3 days. Live/dead assay (Yeasen biotech, 40747ES76) was used to detect cell viability in Sp‐MSCs, and confocal microscopy (Zeiss, Germany) was used.^[^ ^77^ ^]^

### Immunofluorescence

Sp‐MSCs were stained with CD73 (BD Pharmingen mouse anti‐human CD73, 561 254), CD90 (BD Pharmingen mouse anti‐human CD90, 555 595), and DAPI (Antgene, ANT063). A confocal microscope was used for scanning.^[^ ^12^ ^]^

### Scanning Electron Microscopy (SEM)

Sample was fixed in 2.5% glutaraldehyde fixative, dewatering, and drying. Specimens were attached to metallic stubs using carbon stickers and sputter‐coated with gold for 30 s. Images were taken using a scanning electron microscope (Hitachi, SU8100).^[^ ^78^ ^]^

### Flow Cytometry

MSC surface markers were detected using BD Stemflow Human MSC Analysis Kit (BD Biosciences). Sp‐MSCs were dissociated into individual cells. Cell suspensions were stained via flow cytometry for MSC‐positive markers (CD44, CD73, CD90, and CD105) and were detected using corresponding antibodies on BD Accuri 6 flow cytometer (BD Biosciences).^[^ ^12^ ^]^

### Preparation of PRF

The preparation methods of PRF have been summarized in relevant studies and differential centrifugation method was selected to prepare PRF.^[^ ^23^, ^44^, ^79^, ^80^ ^]^ Mouse (C57BL, female, 6–8w) blood from orbital vein was collected into a vacuum anticoagulant blood collection tube. And immediately placed in a centrifuge (Eppendorf Centrifuge 5810R, Germany) and centrifuged at 700 × *g*, 4 °C for 8 min, and then 1200 × *g*, 4 °C for 5 min. Finally, a syringe was used to extract the lower 1/2layer solution of the plasma layer. 0U thrombin (Biosharp 9002‐04‐4) per milliliter was added, placed at 25 °C, and left to form platelet‐rich fiber (PRF) gel. PRF was immediately used for experimentation or storage at −80 °C environment.

### Gelation Time of PRF and PRF Combined with Sp‐MSCs

After preparation of PRF, 400 Sp‐MSCs were added into 1 mL PRF immediately and stayed at 25 °C. The state of PRF until PRF coagulation was observed and coagulation time was recorded.

### Viscosity Measurement

A rheogoniometer (Haake Mars40, Germany) was used to test the viscosity of PRF/PRF+Sp‐MSCs. PRF and PRF+Sp‐MSCs were prepared for 2 mL each sample following the aforementioned method. PRF/PRF+Sp‐MSCs gelation was obtained and put in a rheogoniometer. Control temperature rose from 4.0 to 37.0 °C, heating rate: 1.0 °C min^−1^ and the viscosity was measured.

### Growth Factor Determination

Enzyme‐linked immunosorbent assay (ELISA) was used to measure the content of growth factors in PRF. The fiber block tissue was ground in PRF and mixed it with the plasma tissue in PRF. It was diluted to a certain multiple and ELISA kits were used to detect PDGF, EGF, VEGF, and TGF‐β in it, then analyzed according to the ELISA kit (Jianglaibio, JL18341, JL20082, JL45919, JL10101).

### Stem Cells Proliferation Activity

1000 MSCs were inoculated on 96‐well plates with 100 µL complete medium, HA group added 10% HA and PRF group added 10% PRF, and incubated with CCK‐8 reagent for 2h. Optical density (OD) was measured at 450 nm, and all tests were repeated three times.

### PRF Protects Sp‐MSCs from Oxidative Stress

Hyaluronic acid (HA) (Hyprojoint, Bloomage Biotech) was used as a regular scaffold carrier. After collection Sp‐MSCs, 200 Sp‐MSCs were coated with 0.5 mL HA/PRF, and the control group was not coated with gel. Store Sp‐MSCs at 25 °C in MSC culture medium. Oxidative stress group added 500 µm H_2_O_2_.^[^ ^27^, ^28^ ^]^ After 2 and 4 days, live/dead and DCFH‐DA (MX4802, MKbio, Shanghai) staining and photographed under a confocal microscope for recording.

### Sp‐MSCs Migration Capability

Before Sp‐MSCs were inoculated on the cell culture plate, HA/PRF gel was spread on the culture plate, and the control group was added with the same amount of blank medium control. After 24 h, photographed under an inverted microscope and the area of Sp‐MSCs was calculated that had crawled out of the spheroids.

### Sp‐MSCs Secretion Assay

Sp‐MSCs were collected and cultured into 24‐well plate at a density of 25 sp cm^−2^. 1 mL MSC culture medium was added, and PRF group added 10% PRF. After 24 h, the MSC culture medium was replaced by 1 mL α‐MEM with 10% FBS. Then the α‐MEM was collected. The concentration of EGF, VEGF, and TGF‐β was measured by ELISA.

### In Vitro Vascular Formation Ability Experiment

Sp‐MSCs were collected and cultured into six‐well plate at a density of 25 sp cm^−2^. And covered with 400 µL PRF. After co‐culturing PRF with Sp‐MSCs for 48 h, the conditioned culture medium (CM) was collected.^[^ ^77^ ^]^

### HUVECs Proliferation Activity

1000 HUVECs were inoculated on 96‐well plates and added 10% each group's CM, and cell viability was measured using CCK‐8 reagent.

### HUVECs Migration Assessment

Six‐well plates were inoculated with 6 × 10^5^ HUVECs per well, and after the cells grew to full size, they were scratched with a 200 µL pipette tip using a straightedge aid, and incubated with different CM. 0 and 12 h were photographed under the inverted fluorescence microscope.^[^ ^81^ ^]^

### HUVECs Tube Formation Assessment

60 µL matrix gel was added to 96‐well plates and incubated at 37 °C for 30 min. Then 5000 HUVECs were inoculated on the surface and 100 µL medium supplemented with 20 µL of different CM was added to each well. 4 h of incubation at 37 °C was used to take pictures using the inverted fluorescence microscope. Evaluating the ability to promote angiogenesis by measuring the length of blood vessel formation and the number of nodes in each group.^[^ ^81^, ^82^ ^]^

### Transcriptomics Analysis

Oxidative stress modeling treatment was the same as before. After a culture of 2 days, RNA was collected from each group. Output Illumina platform. Gene expression levels were normalized by calculating TPM (Transcripts Per Kilobase of exon model per Million mapped reads). DESeq2 was used to perform the differentially expressed genes (DEGs) analysis.^[^ ^83^ ^]^ DEGs were used for subsequent Kyoto Encyclopedia of Genes and Genomes (KEGG) enrichment analyses were performed using the Bio conductor packages edgeR.^[^ ^84^ ^]^ Hierarchical cluster analysis was performed using clusterProfiler and heatmap generation was performed using R4.2.1.^[^ ^85^ ^]^

### RT‐qPCR

Total RNA was extracted from tissues and cells using Trizol (Invitrogen, USA) and reverse transcribed to cDNA by using a Reverse Transcription Kit (TOYOBO, OSAKA, Japan). The RNA transcript levels were analyzed using a QuantStudio3 real‐time PCR system (Thermo Fisher, USA) and normalized to GAPDH. Primers used in RT‐qPCR are listed in Table S1 (Supporting Information).^[^ ^39^, ^40^, ^41^, ^42^, ^86^, ^87^, ^88^, ^89^ ^]^

### Animal Experiments

All experimental animals used in this study were provided by the Department of Laboratory Animals of the Central South University (Changsha, China) and maintained under specific pathogen‐free conditions. The Research Ethics Committee of Central South University approved all experimental protocols in this study (csu‐2023‐0196).

Construction of a Diabetic Wound Model: All *db/db* mice (female, 6–8w) were subjected to a period of fasting that lasted for 4 h.^[^ ^90^ ^]^ After anesthesia, a circular skin wound (diameter = 10 mm) was built on the dorsum of the mouse using a surgical scissor, then covering silver ion antibacterial gel. Subsequently, cover the wound site with PRF+Sp‐MSCs (100 µL PRF dispersed in 100µL PRF)/PRF (100 µL PRF)/Sp‐MSCs (200Sp‐MSCs dispersed in 100 µL HA), and leave the control group untreated. Ultimately, observations and photography of the wound‐healing process took place on days 1, 5, 9, 12, and 15.^[^ ^91^, ^92^ ^]^

Ex Vivo Imaging Analysis: To visualize cells, MSCs were labeled with DiR iodide (1,1′‐Dio ctadecyl‐3,3,3′,3′‐Tetramethylindotricarbocyanine iodide) (Bergolin Biotechnology, Dalian, China) for 30 min at 37 °C, then forming Sp‐MSCs. After transplantation of Sp‐MSCs, the wound area was obtained for imaging by an AniView100X multimode imaging system (Biolight Biotechnology, Co., Ltd., China). Measure and analyze the fluorescence intensity of the wound to reflect the survival status of Sp‐MSCs in the wound.

Wound Healing Mechanism Study: *db/db* mice were euthanized at predetermined time points, and dissected to obtain regenerated dorsal skin, and placed in a 4% paraformaldehyde solution for fixation. The fixed tissue sections were stained with hematoxylin and eosin (H&E), Masson, Sirius red staining, CD34, αSMA, GAP43, VEGF, CD206, TNF‐α, H3Cit.

### Image Analysis and Rendering

ImageJ (National Institutes of Health, America) was used for image correlation measurement and quantitative analysis.

### Statistical Analysis

Standard statistical analysis was performed using GraphPad Prizm 9.5.0. The statistical details in the graphics are presented as the mean ± standard deviation. The Student's *t*‐test was used for the determination of statistical significance between the two groups (*n* = 2). The ANOVA test was used for the determination of statistical significance between multiple groups (*n* ≥ 3). And the result was deemed statistically significant at *p* < 0.05, (where “*” signifies *p* < 0.05, “**” signifies *p* < 0.01, “***” signifies *p* < 0.001, “****” signifies *p* < 0.0001, and “ns” signifies *p* > 0.05).

## Conflict of Interest

The authors declare no conflict of interest.

## Author Contributions

J.Z., W.X., Y.X., D.S., and D.W. conducted experiments on in vitro and in vivo investigation. J.Z., W.X., and D.W. were responsible for animal experiments. J.Z. and D.W. analyzed the data. J.Z., D.W., and S.L. were responsible for guiding experiments. J.Z. and D.W. wrote the manuscript. All authors read and approved the final manuscript.

## Supporting information



Supporting Information

## Data Availability

The data that support the findings of this study are available on request from the corresponding author. The data are not publicly available due to privacy or ethical restrictions.
