# Correction to “Accurate Delivery of Mesenchymal Stem Cell Spheroids With Platelet‐Rich Fibrin Shield: Enhancing Survival and Repair Functions of Sp‐MSCs in Diabetic Wound Healing”

**DOI:** 10.1002/advs.77324

**Published:** 2026-08-24

J. Zhang, W. Xu, Y. Xiao, D. Su, Y. He, H. Yang, Y. Xie, X. Wang, R. Xu, S. Lei, and D. Wu, “Accurate Delivery of Mesenchymal Stem Cell Spheroids With Platelet‐Rich Fibrin Shield: Enhancing Survival and Repair Functions of Sp‐MSCs in Diabetic Wound Healing,” *Advanced Science* 12, no. 25 (2025): 2413430, https://doi.org/10.1002/advs.202413430.

**In the published article, some instances of the term “autologous” have been corrected to “allogeneic”, and some source qualifiers have been removed**. These inaccuracies arose due to an oversight in terminology during manuscript preparation. In the described experiments, due to insufficient blood volume in db/db mice, we used C57BL mice as PRF donors. Therefore, the PRF used in this study was allogeneic, not autologous. We have corrected the terminology in sections describing the experimental methods and results, and removed source qualifiers in sections discussing general PRF principles and clinical applications to maintain scientific accuracy and avoid ambiguity.

The relevant sections of the article have been amended accordingly.

**1. Abstract**:

 from “[…] the use of autologous plasma‐rich platelet fibrin (PRF) as […]”

 to “[…] the use of allogeneic plasma‐rich platelet fibrin (PRF) as […]”.

**Reason**: The abstract describes the experimental approach of this study. Since PRF was prepared from allogeneic C57BL mice in this experiment, “allogeneic” is the correct term.

**2. Discussion, Paragraph 1**:

 from “[…], our study employed PRF gel, derived from autologous plasma, […]”

 to “[…], our study employed PRF gel, derived from allogeneic plasma, […]”.

**Reason**: This sentence describes the specific experimental approach of this study. Since PRF was prepared from allogeneic C57BL mice, “allogeneic” is the correct term.

**3. Discussion, 3.1, Paragraph 2**:

 from “Then we prepared PRF gel from autologous plasma by differential centrifugation […]”

 to “Then we prepared PRF gel from allogeneic plasma by differential centrifugation […]”.

**Reason**: This sentence describes the specific experimental method. Since PRF was prepared from allogeneic C57BL mice, “allogeneic” is the correct term.

**4. Discussion, 3.1, Paragraph 2**:

 from “Furthermore, PRF is entirely derived from the autologous plasma of patients has high biocompatibility, […]”

 to “Furthermore, PRF derived from the autologous plasma of patients aims to achieve high biocompatibility, […]”.

**Reason**: The original sentence contains a grammatical error with dual predicates (“is derived” and “has”). Additionally, the assertion that PRF “entirely” derived from autologous plasma “has” high biocompatibility presents an absolute claim that is inconsistent with general clinical practice, where PRF may be prepared from either autologous or allogeneic sources. The revision corrects the syntax, removes the absolute qualifier “entirely,” and replaces “has” with “aims to achieve”, thereby presenting a purpose‐driven description that is more appropriate for discussing clinical applications and avoids conflating general practice with the specific allogeneic source used in this experiment.

**5. Discussion, 3.4, Paragraph 1**:

 from “[…], the autologous plasma from which it is derived imposes certain constraints.”

 to “[…], the allogeneic plasma from which it is derived imposes certain constraints.”

**Reason**: This sentence refers to the specific plasma source used in this experiment. Since C57BL mice were used as PRF donors, “allogeneic” is the correct term.

**6. Discussion, 3.4, Paragraph 4**:

 from “Autologous plasma gel emerges as an accessible, […]”

 to “Allogeneic plasma gel emerges as an accessible, […]”.

**Reason**: This sentence refers to the specific plasma gel used in this experiment. Since PRF was prepared from allogeneic C57BL mice, “allogeneic” is the correct term.

**7. Discussion, 3.4, Paragraph 4**:

 from “The utilization of biomaterial gels derived from autologous plasma paves novel avenues […]”.

 to “The utilization of biomaterial gels derived from allogeneic plasma paves novel avenues […]”.

**Reason**: This sentence refers to the specific plasma gel used in this experiment. Since PRF was prepared from allogeneic C57BL mice, “allogeneic” is the correct term.

″We have corrected “autologous” to “allogeneic” in accordance with the experimental methods and results of this study, which differ from the clinical practice of using ‘autologous’ materials for biocompatibility and immune compatibility. Sections containing “autologous” that cite relevant literature or refer to the clinical practice of using “autologous” materials remain unchanged for accuracy of description.

We apologize for this oversight and any confusion it may have caused. This correction does not affect the main findings, conclusions, or scientific validity of the article.

The authors apologize for these errors and for the inconvenience they may have caused. All authors agree with the above editing.

