# Supplementary material for: Accurate Delivery of Mesenchymal Stem Cell Spheroids With Platelet‐Rich Fibrin Shield: Enhancing Survival and Repair Functions of Sp‐MSCs in Diabetic Wound Healing
Source: Adv Sci (Weinh). 2025 May 28;12(25):2413430. doi: 10.1002/advs.202413430 (PMC12225000; doi:10.1002/advs.202413430)
Supplement: Supplementary file 1 — Supporting Information [file ADVS-12-2413430-s001.docx]

**Accurate Delivery of Mesenchymal Stem Cell Spheroids with Platelet-Rich Fibrin Shield: Enhancing Survival and Repair Functions of Sp-MSCs in Diabetic Wound Healing**

Jinglve Zhang^#1, 2^, Wenqing Xu^1, 2^, Yutian Xiao^1, 2^, Dingheng Su^1, 2, 3^, Yusheng He^4, 5^, Huohong Yang^4, 5^, Yixin Xie^4, 5^, Xiaofang Wang^4, 5^, Ren-He Xu^6^, Shaorong Lei*^1, 2^, Dingyu Wu*^1, 2^

^1^ Department of Plastic Surgery, Xiangya Hospital, Central South University, 87 Xiangya Road, Changsha, Hunan, China, 410008.

^2^ National Clinical Research Center for Geriatric Disorders, Xiangya Hospital, Changsha, Hunan, China, 410008.

^3^ Xiangya School of Medicine Central South University, Changsha, China, 410083.

^4^ ImStem Biotechnology, Inc., 400 Farmington Avenue R1808, Farmington, CT 06030, USA.

^5^ Zhuhai Hengqin ImStem Biotechnology Co., Ltd, Hengqin New District Huandao Donglu 1889 Building 3, Zhuhai 519000, Guangdong, China.

^6^ Ministry of Education Frontiers Science Center for Precision Oncology, Center of Reproduction, Development & Aging, and Institute of Translational Medicine, Faculty of Health Sciences, University of Macau, Taipa, Macau, China.

*Correspondence:

Shaorong Lei, leishaorong@csu.edu.cn

Dingyu Wu, 4011609@csu.edu.cn

## Supplemental Materials


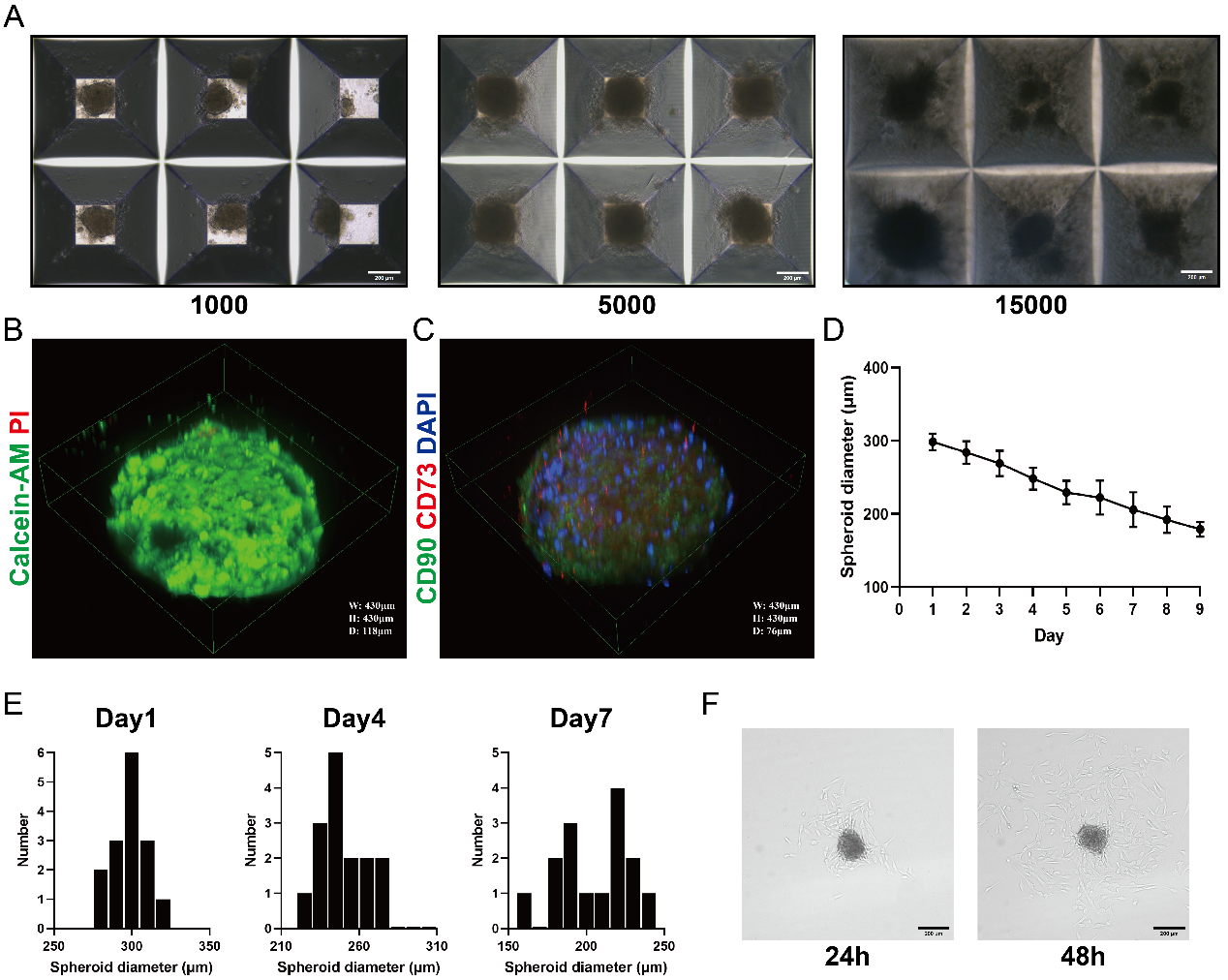


FigureS1: (A) Image of 1000/5000/15000 cells/spheroid Sp-MSCs in cell spheres forming plate. 5000 cells/spheroid had the most stable size (B) The confocal image of live/dead staining. (C) The confocal image of Stem cell markers (CD90, green; CD73, red; DAPI, blue) of the cells in the Sp-MSCs. (D) The diameter of Sp-MSCs gradually decreased from day 1 to day 9, stored at 25 ℃ in MSC culture medium (n=15). (E) histogram of Sp-MSCs’ diameter at day1, day4, day7. (F) Sp-MSCs after 7 days of storage, image of recovery and crawling out at 24h and 48h.


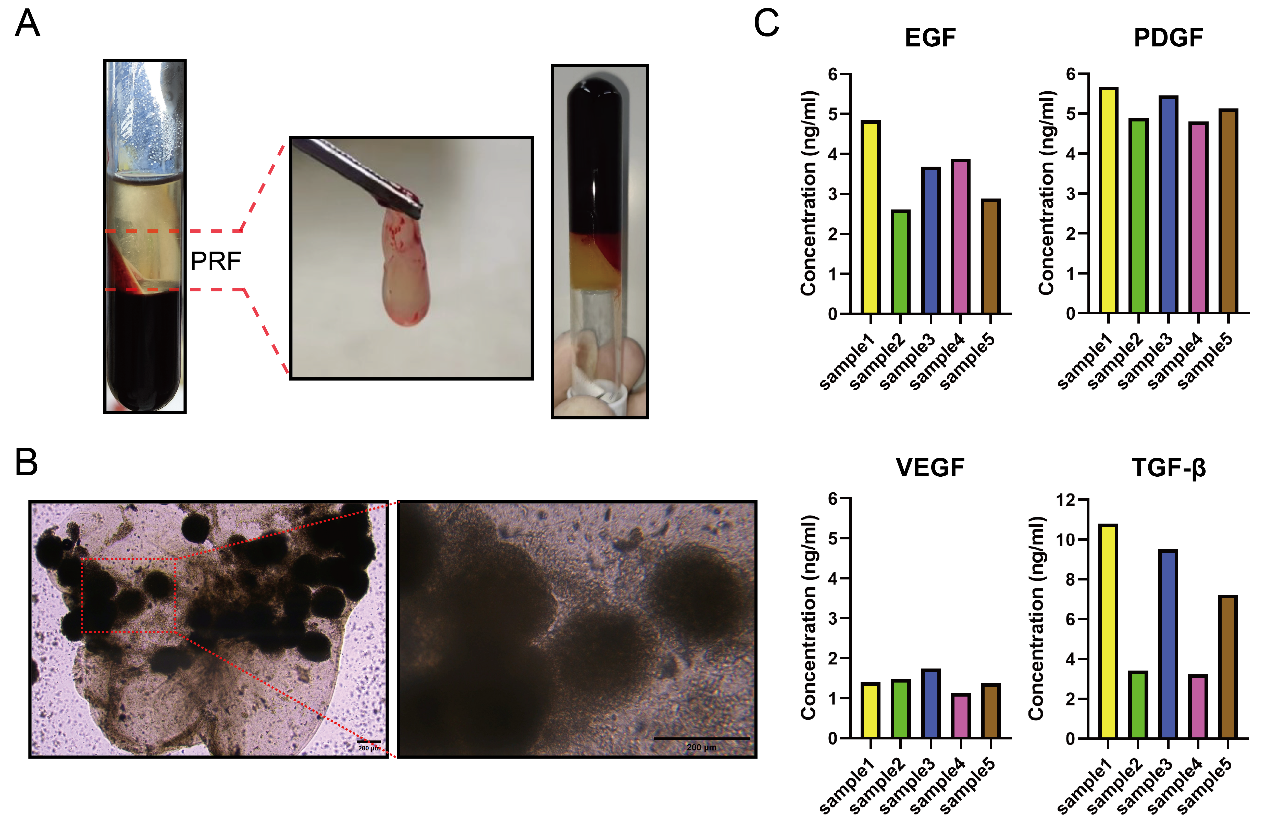


FigureS2: (A) Image of PRF prepared by differential centrifugation method. (B) Image of PRF coated Sp-MSCs (scale bar=200μm)_._ (C) ELISA analysis of EGF, PDGF, VEGF, and TGF-β of 5 PRF samples.


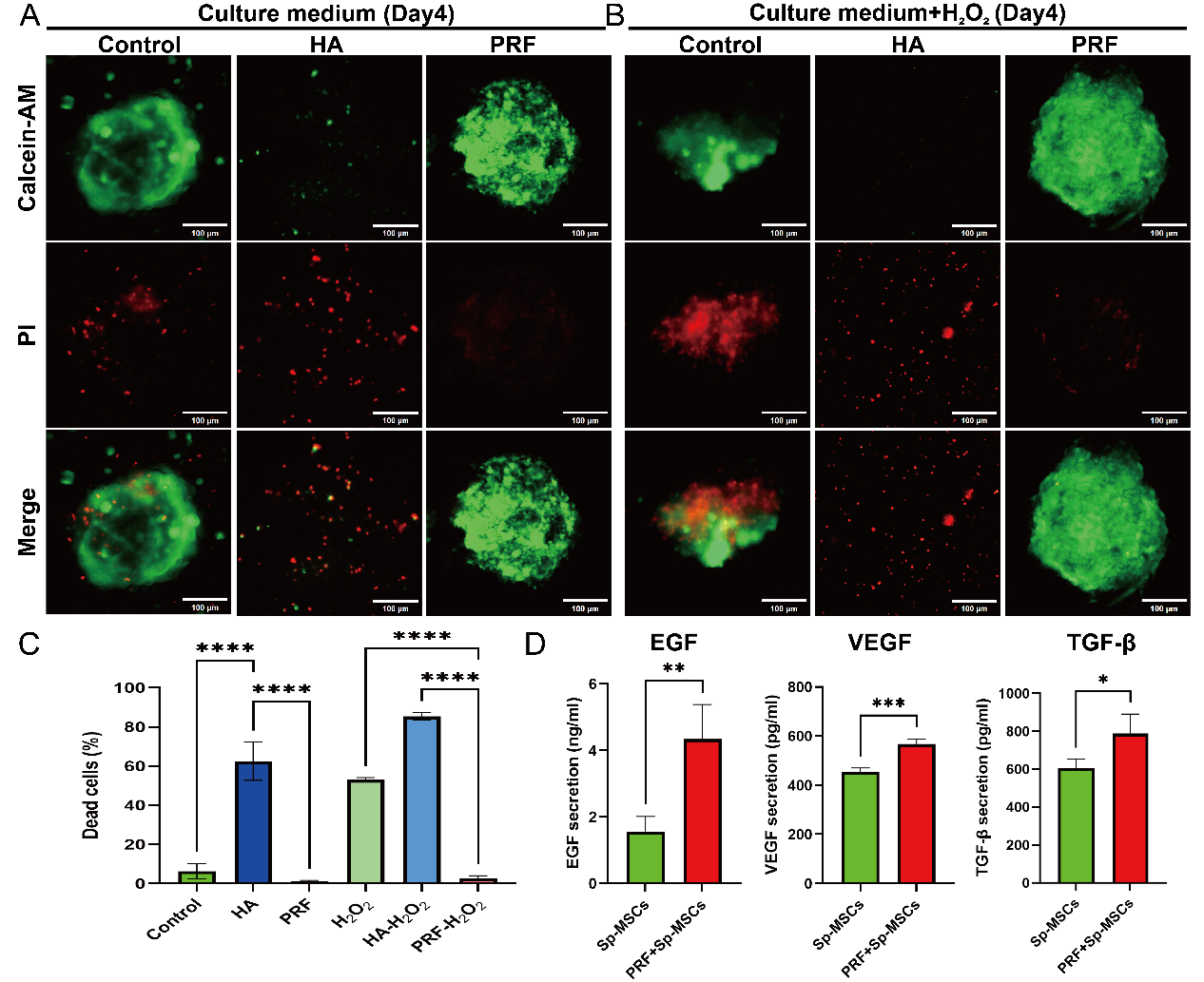
FigureS3: (A) Live/Dead staining showing the cell viability of exposed Sp-MSCs and encapsulated Sp-MSCs in HA/PRF at 4 days during in vitro culture. (B) Live/Dead staining showing the cell viability of exposed Sp-MSCs and encapsulated Sp-MSCs in HA/PRF at 4 days during in vitro culture after treatment with 500μM H_2_O_2_ (Scale bar=100μm). (C) Quantitative analysis based on live/dead staining of the cell viability (n=3, **** p < 0.0001). (D) The secretion of EGF, VEGF, and TGF-β of Sp-MSCs and PRF coated Sp-MSCs, analyzed by ELISA (n=4, * p < 0.05, ** p < 0.01, *** p < 0.001).


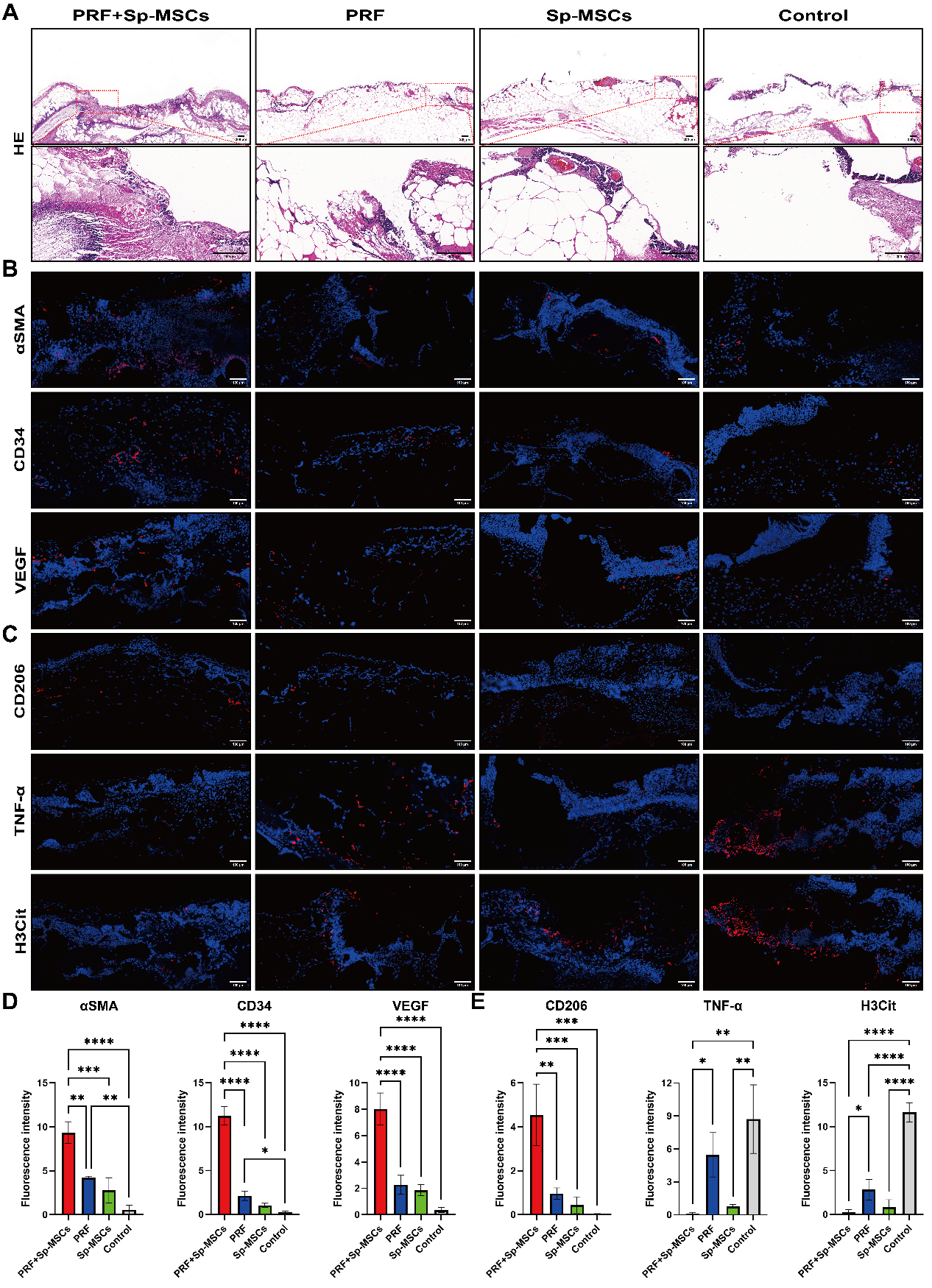


FigureS4: The effect of PRF combined with Sp-MSCs on tissue regeneration and immune regulation on day 5. (A) H&E staining of wound tissues at day 5 (Scale bar=200μm). (B) Immunofluorescence of αSMA, CD34 and VEGF showed angiogenesis (Scale bar=100μm). (C) Immunofluorescence of CD206 showed accumulation of M2 macrophages, TNF-α showed pro-inflammatory factors, and H3Cit showed neutrophil recruitment at the edge of wound on day 5 (Scale bar=100μm). (D, E) Quantitative analysis based on immunostaining of αSMA, CD34, VEGF, CD206, TNF-α, H3Cit (n=3, * p < 0.05, ** p < 0.01, *** p < 0.001, **** p < 0.0001).

Table1: Primers sequence for RT-qPCR

| **Names** | **Sequences (5’-3’)** |
| --- | --- |
| *Bax*(F) | TGGCAGCTGACATGTTTTCTGAC |
| *Bax*(R) | TCACCCAACCACCCTGGTCTT |
| *Bcl2*(F) | TCGCCCTGTGGATGACTGA |
| *Bcl2*(R) | CAGAGACAGCCAGGAGAAATCA |
| *Ki67*(F) | TCCAGACGCCAAAATAAGACTG |
| *Ki67*(R) | TCCATCTCTGGGGAGGTCTTC |
| *VEGF*(F) | AGCCTTGCCTTGCTGCTCTAC |
| *VEGF*(R) | TGATGATTCTGCCCTCCTCCTT |
| *TGFβ*(F) | TCGCCAGAGTGGTTATCTT |
| *TGFβ*(R) | TAGTGAACCCGTTGATGTCC |
| *Integrin-β1*(F) | GCAACGCATATCTGGAAACT |
| *Integrin-β1*(R) | CAAAGTGAAACCCAGCATCC |
| *β-catenin*(F) | GCTGACCAAACTGCTAAATGACGA |
| *β-catenin*(R) | TGTAGGGTCCCAGCGGTACAA |
| *FZD2*(F) | GCGTCTTCTCCGTGCTCTAC |
| *FZD2*(R) | CTGTTGGTGAGGCGAGTGTA |
| *PORCN*(F) | TTCCCGTACTTCATCCCCCT |
| *PORCN*(R) | CCACCTTACCATGGTGCCC |
| *Axin*(F) | AGAAATGCATCGCAGTGTGAAG |
| *Axin*(R) | GGTGGGTTCTCGGGAAATG |
| *GAPDH*(F) | AGACAGCCGCATCTTCTTGT |
| *GAPDH*(R) | CTTGCCGTGGGTGAGTCAT |
